# Ezrin Functions as a Key Mechanosensitive Effector to Preserve Cell Structure and Promote Survival and Metastasis of Lung Cancer Cells during Circulation

**DOI:** 10.7150/ijbs.127134

**Published:** 2026-09-03

**Authors:** Xiaoying Zhang, Muya Zhou, Koukou Li, Mingheng Yuan, Haibo Tong, Kathy Qian Luo

**Affiliations:** 1 Department of Biomedical Sciences, Faculty of Health Sciences, University of Macau, Taipa, Macao SAR, China.; 2 Ministry of Education Frontiers Science Center for Precision Oncology, University of Macau.

**Keywords:** shear stress, lung cancer, Ezrin phosphorylation, CD44, ROS, survival and metastasis

## Abstract

Metastasis remains the leading cause of cancer-related mortality, a process in which circulating tumor cells (CTCs) play a central but incompletely understood role. Although most CTCs are rapidly eliminated during circulation through detachment-induced anoikis and fluid shear stress (SS)-triggered apoptosis, a subset of CTCs can survive these hostile conditions; however, the underlying mechanisms remain poorly defined. To investigate this, we developed a microfluidic circulatory system that mimics physiological SS and performed transcriptomic profiling of A549 lung cancer cells under SS, attached, and static suspension conditions. Through this approach, we identified the ROS-CD44-Ezrin axis as a potential mechanosignaling module that may contribute to CTC survival under shear stress. In this cascade, SS-induced ROS and CD44 converge to activate Ezrin via phosphorylation at Thr567. Once activated, Ezrin appears to coordinate its two canonical functions-membrane-cytoskeleton reinforcement and pro-survival signaling via PI3K/AKT/Bcl-2 and NF-κB/p38/JNK pathways-within this specific mechanobiological context to preserve cell integrity and limit apoptosis. Our findings suggest that these well-established molecular functions are differentially engaged under shear stress, revealing a context-dependent survival mechanism that may offer opportunities for therapeutic exploration, pending further validation in more clinically relevant models.

## Introduction

Cancer remains one of the most life-threatening diseases worldwide, with lung cancer standing out as the primary cause of cancer deaths 1. Metastasis, the spread of cancer cells from the primary tumor to distant organs, is the leading cause of cancer-related fatalities, and circulating tumor cells (CTCs) are considered to play a critical role in this process 2. Research has shown that elevated levels of CTCs in the blood are associated with poorer clinical outcomes in various cancers, including breast 3, lung 4, colorectal 5, and prostate cancer 6. Despite the fact that millions of cancer cells are shed into the bloodstream, only fewer than 0.02% of CTCs could successfully establish metastatic colonies in distant tissues 7-9. Understanding the mechanisms that enable these rare CTCs to survive in the harsh circulatory environment may inform future strategies aimed at preventing metastasis in cancer patients.

When CTCs enter the bloodstream, they face three significant survival challenges. The first is anoikis, a form of programmed cell death triggered by the loss of attachment to their original extracellular matrix and neighboring cells. Additionally, CTCs must evade attacks from the immune system. Lastly, they must withstand the mechanical forces in the circulatory system, particularly hemodynamic shear stress (SS), which can damage or destroy cells as they navigate through the bloodstream 10-13. Overcoming these barriers is essential for the survival and successful colonization of CTCs 14.

The magnitude of SS within the human circulatory system varies significantly depending on the vascular environment. In veins, SS typically ranges from 0.5 to 4 dynes/cm², while in arteries, it can reach much higher levels, ranging from 4 to 30 dynes/cm² 15-18. These varying levels of SS exert distinct effects on CTCs. Lower SS, such as those found in venous circulation, appears to have minimal detrimental impact on CTCs, while elevated SS levels characteristic of arterial flow (12 to 30 dynes/cm²) pose a significant threat to CTCs, leading to a marked increase in apoptosis 19, 20. Elucidating the mechanisms of how CTCs withstand SS is therefore of considerable interest for cancer therapy.

To study the impacts of SS on CTCs, researchers have developed various *in vitro* models to simulate blood flow conditions. These include methods such as stirring cell suspensions 21, rotating cells 22, using cone and plate viscometers 23, 24, syringes and needles 25, 26, parallel plate flow chambers 27, and microfluidic devices 28. Our group previously designed a microfluidic circulatory system to mimic SS *in vitro*, and revealed that CTCs tend to form clusters to protect themselves from SS damage and contribute to metastatic potential, which was facilitated by the upregulation of desmosomal proteins desmocollin-2 and plakophilin-1 upon circulatory treatment 29.

While earlier studies often overlooked the dual challenges of detachment and SS that CTCs face in circulation, we previously analyzed lung cancer cells under adherent, suspended and circulatory conditions and demonstrated that SS specifically promoted CTC metastasis through upregulation of mesotrypsin (PRSS3) and protease-activated receptor 2 (PAR2) 30. In that study, we also observed SS-induced cell death in CTCs; however, the mechanisms by which a subset of CTCs survived SS damage remain unclear.

To address this, we further analyzed our RNA-sequencing data to identify key protective genes in SS-resistant CTCs. In this study, our data suggest the ROS-CD44-Ezrin axis as a potential integrated mechanosignaling module in SS-resistant CTCs. While the individual functions of Ezrin as a membrane-cytoskeleton linker and signaling scaffold are well established, our findings suggest that under shear stress conditions, these canonical roles may be coordinately engaged via ROS-induced signaling and CD44 cooperation. Specifically, SS triggers Ezrin phosphorylation at Thr567, enabling it to couple two context-dependent outputs: stabilization of membrane-cytoskeleton integrity through CD44 interaction, and concurrent activation of PI3K/AKT/Bcl-2 and NF-κB /p38/JNK survival pathways.

Together, these observations point to a context-specific mechanoadaptive role for the ROS-CD44-Ezrin axis in promoting CTC survival under shear stress. Further investigation will be required to determine whether this mechanism operates in more clinically relevant settings and whether it may offer opportunities for therapeutic intervention.

## Materials and Methods

### Cell lines and culture conditions

The non-small cell lung cancer (NSCLC) cell line A549 and the human embryonic kidney cell line 293T were procured from the American Type Culture Collection (ATCC). These cells were maintained in Dulbecco's Modified Eagle's Medium (DMEM; #12100046, Thermo Fisher Scientific, USA). Additionally, the NSCLC cell line H1975 was kindly provided by Prof. Joong Sup Shim from the Faculty of Health Sciences at the University of Macau, Macau, China, and cultured in Roswell Park Memorial Institute (RPMI) 1640 medium (#31800022, Thermo Fisher Scientific, USA).

All culture media were supplemented with 10% fetal bovine serum (FBS; #10270-106, Gibco, USA) and 1% penicillin-streptomycin antibiotic solution (#15140122, Thermo Fisher Scientific, USA) to ensure optimal growth conditions. A549-C3 and A-SSP6 cells were established using previously published protocols 29.

### Suspension culture and microfluidic circulatory system setup

To achieve suspension conditions, cancer cells were trypsinized and resuspended at a density of 2 × 10⁵ cells per mL in standard culture medium using ultralow attachment 6-well plates (#3471, Corning, USA). The cells were then incubated in a humidified CO₂ incubator at 37 °C for specified time periods and collected for analysis.

The microfluidic circulatory system was custom-designed based on methodologies established in prior studies 29, 30. Briefly, the system consisted of several key components: a reservoir equipped with a cotton filter to minimize contamination and medium evaporation, a connecting tube, a peristaltic pump (Ismatec, Germany) to generate pulsatile shear flow, and a circulatory tube with a diameter of 500 μm and a length of 1.5 m. The shear stress (SS) levels within the system were calculated using Poiseuille's equation: 𝜏 = 4Q𝜂∕𝜋R³, where 𝜏 represents the shear stress (in dyne cm⁻²), Q is the flow rate (in cm³ s⁻¹), 𝜂 is the dynamic viscosity of the fluid (0.012 dyne s cm⁻²), and R is the radius of the tube (250 μm). In this study, the primary focus was on a 15 dynes cm^-2^ (SS15), as it approximates the average shear stress found in human arteries.

Prior to use, all system components were sterilized with 70% ethanol and rinsed three times with Milli-Q water. To prevent cell adhesion to the tubing, the entire system was coated with 1% Pluronic F-127 (#P2443, Sigma-Aldrich, Germany). Cancer cells were trypsinized, resuspended at a density of 2 × 10⁵ cells per mL in culture medium, and introduced into the reservoir. Following the circulatory treatment, cells were harvested, transferred to a confocal dish, and imaged using a Carl Zeiss microscope (10× objective, Axio Observer, Germany) or subjected to other analysis.

### MTT assay for cell viability

Cells were harvested at three different conditions: 0 h (baseline) and shear stress (SS). A volume of 100 μL of cell suspension was seeded into each well of a 96-well plate. Then 10 μL of MTT solution (#M2128, Sigma-Aldrich, Germany) was added to each well, and the plates were incubated for 4 hours at 37 °C to allow formazan crystal formation. After incubation, 100 μL of a solubilization solution containing 10% sodium dodecyl sulfate (SDS) and 0.01 M HCl was added to each well to dissolve the crystals. The plates were then incubated overnight at 37 °C to ensure complete solubilization. The absorbance of each well was measured at 595 nm using a microplate reader (PerkinElmer VICTOR X3, USA) to quantify cell viability.

### Transwell migration assay

Cells were trypsinized, and resuspended in serum-free DMEM. A total of 10,000 cells in 100 μL of serum-free medium were seeded onto the upper chamber of a Transwell insert (#3422, Corning, USA). The lower chamber was filled with 600 μL of complete culture medium containing 10% fetal bovine serum to act as a chemoattractant. The plates were then incubated in a humidified CO₂ incubator at 37 °C for the specified duration to allow cell migration.

After incubation, non-migrated cells on the upper surface of the membrane were carefully removed using a cotton swab. Migrated cells on the lower surface of the membrane were fixed with 4% paraformaldehyde (PFA; #158127, Sigma-Aldrich, Germany) for 15 minutes at room temperature. The fixed cells were then stained with 0.5% crystal violet (#C6158, Sigma-Aldrich, Germany) for an additional 15 minutes to visualize migrated cells. The membrane was then washed with Milli-Q water, excised from the Transwell insert and mounted onto a glass slide using a mounting medium (#06522, Sigma-Aldrich, Germany). Migrated cells were imaged and quantified under a light microscope to assess cell migration capability under different experimental conditions.

### Colony formation assay

To assess clonogenic potential, 1,000 cells per well were seeded into 6-well plates and cultured in complete growth medium for 7 days under standard culturing conditions (37 °C, 5% CO₂). After the incubation period, the culture medium was carefully aspirated, and the cells were gently washed with phosphate-buffered saline (PBS) to remove debris. The colonies were then fixed and stained with 0.5% crystal violet solution for 15 minutes at room temperature. Excess stain was rinsed off with distilled water, and the plates were air-dried. Colony images were captured using a digital camera. The number of colonies and their respective areas were quantified using ImageJ software (National Institutes of Health, USA).

### RNA extraction and quantitative PCR (qPCR)

Total RNA was isolated from cells using TRIzol reagent according to the manufacturer's instructions. Reverse transcription was performed using the iScript cDNA Synthesis Kit (#1778890, Bio-Rad, USA) to generate complementary DNA (cDNA). For qPCR analysis, reactions were carried out using the iTaq Universal SYBR Green Supermix (#1725122, Bio-Rad, USA) on a real-time PCR system. Gene-specific primers, designed and validated for target genes, were used for amplification (primer sequences are provided in Supplementary Table 1).

### Western blot analysis

Cells were harvested, washed once with PBS, and lysed using radioimmunoprecipitation assay (RIPA) buffer supplemented with protease and phosphatase inhibitor cocktails (Sigma-Aldrich, Germany). Protein concentrations were quantified using the Bio-Rad protein assay. Equal amounts of protein were resolved by SDS-polyacrylamide gel electrophoresis (SDS-PAGE) and subsequently transferred onto nitrocellulose membranes (Bio-Rad, USA).

Membranes were blocked with 5% blotting-grade blocker (#1706404, Bio-Rad, USA) for 1 hour at room temperature to prevent nonspecific binding. They were then incubated with primary antibodies overnight at 4 °C. After washing, membranes were probed with horseradish peroxidase (HRP)-conjugated secondary antibodies for 1 hour at room temperature. Detailed information on the primary and secondary antibodies used is provided in Supplementary Table 2.

Protein bands were detected using Clarity Western ECL Substrate (#1705061, Bio-Rad, USA) and visualized with a ChemiDoc Touch Imaging System (Bio-Rad, USA). Band intensities were quantified using ImageJ software (National Institutes of Health, USA), and statistical significance was determined using Student's t-test.

### Immunofluorescence staining

Sterilized glass coverslips were coated with poly-D-lysine hydrobromide (#P7886, Sigma-Aldrich, Germany) for 30 minutes to enhance cell adhesion. Cancer cells were collected and seeded onto the coated coverslips, followed by incubation at 37 °C for 20-30 minutes to allow attachment. Cells were then fixed with 4% PFA for 15 minutes at room temperature and permeabilized with 0.2% Triton X-100 (#T8787, Sigma-Aldrich, Germany) for 15 minutes. To reduce nonspecific binding, cells were blocked with 3% bovine serum albumin (BSA; #4240GR500, BioFroxx, Germany) for 1 hour at room temperature.

Primary antibodies were applied and incubated overnight at 4 °C. After washing, cells were incubated with Alexa Fluor-conjugated secondary antibodies (Invitrogen, USA) for 1 hour at room temperature in the dark. Nuclei were counterstained with Hoechst 33342 (#H3570, Thermo Fisher Scientific, USA) for 10 minutes. Fluorescent images were acquired using a confocal microscope (Carl Zeiss LSM710, Germany), and fluorescence intensity was quantified using ImageJ software (National Institutes of Health, USA).

### Gene knockdown and overexpression

Gene-specific siRNA duplexes were designed and commercially obtained (Ambion). For transfection, Lipofectamine RNAiMAX (Invitrogen) was used. Briefly, each siRNA was diluted in serum-free medium and allowed to complex with the transfection reagent for 20 min at room temperature. The resulting siRNA-lipid complexes were then added to cultured cells and incubated for 48 h under standard conditions. Knockdown efficiency was assessed by qPCR.

Short hairpin RNA (shRNA) sequences were designed by evaluating the H-b index of candidate sequences to ensure optimal knockdown efficiency. The specific targeting sequences for shRNAs are provided in Supplementary Table 3, while the sequences for gene overexpression constructs are detailed in Supplementary Table 4. All shRNA and overexpression vectors were procured from VectorBuilder (USA).

For gene knockdown, lentiviral vectors encoding shRNA constructs were transfected into cells to silence target gene expression. For overexpression studies, cells were transfected with lentiviruses carrying expression plasmids containing the full-length coding sequences of the target genes. Transduction efficiency was confirmed using appropriate controls, and functional assays were performed to validate the effects of gene modulation.

### Lung colony formation assay and orthotopic lung xenograft model

All animal procedures were conducted in compliance with the guidelines approved by the University of Macau Animal Ethics Committee (Approved Protocol IDs: UMARE-025-2017 and UMARE-026-2017).

For the lung colony formation assay, 1 × 10⁶ cancer cells were injected into the tail vein of 6- to 8-week-old female NOD/SCID mice. After 28 days, the mice were euthanized, and lung tissues were harvested for analysis. Lung metastases were visualized and quantified using an Olympus fluorescence microscope (MVX10, Japan). The number of metastatic colonies in the left lungs was counted to assess the metastatic potential of the cancer cells.

For the orthotopic lung xenograft model, cancer cells were resuspended at a concentration of four million cells per 25 μL in PBS and mixed with an equal volume of Matrigel matrix (#354234, Corning, USA). NOD/SCID mice were anesthetized using 1.25% avertin (#T4840-2, Sigma-Aldrich, Germany), and 50 μL of the cell-Matrigel mixture was injected into the right lungs of each mouse using a 30-gauge hypodermic needle. Body weight was monitored weekly as an indicator of overall health. Four weeks post-injection, mice were euthanized, and lung tissues were excised for imaging using the Olympus fluorescence microscope (MVX10, Japan).

### Immunohistochemistry (IHC)

Tissue microarray slides from non-small cell lung cancer (NSCLC) patients were procured from Superchip Company (China). The slides were deparaffinized in xylene and rehydrated through a graded ethanol series. Antigen retrieval was performed using a citrate-based buffer under heat-induced conditions. IHC staining was carried out using an IHC Detection Kit (#ab64264, Abcam, UK) following the manufacturer's protocol.

After staining, nuclei were counterstained with hematoxylin using a Leica ST5020-CV5030 Multistainer-Coverslipper (Germany). The slides were then scanned at high resolution using a NanoZoomer S60 Digital Slide Scanner (#C13210-01, HAMAMATSU, Japan) to obtain whole-slide images for analysis.

IHC scoring was performed by evaluating both the percentage of positively stained cells and the staining intensity, as previously described 31. The combined score provided a semi-quantitative assessment of protein expression levels in the tissue samples.

### ROS detection

A549 and H1975 cells were circulated for different time points and then immediately stained with 10 μM CM-H2DCFDA (DCFDA; #C6827, Thermo Fisher Sci entific, USA) in serum-free fluoro-brite medium (#A1896701, Thermo Fisher Scientific, USA) for 30 min at 37 °C. The images were taken with a Carl Zeiss microscope and analyzed by using ImageJ software.

### Co-immunoprecipitation

To examine the interaction between EZR and CD44 and its regulation by SS and ROS, A549 and H1975 cells were subjected to control, SS treatment, or SS treatment in the presence of an antioxidant propyl gallate (PG). Cells were lysed in NP-40 buffer (20 mM Tris-HCl, pH 7.5, 137 mM NaCl, 10% glycerol, 1% NP-40, 2 mM EDTA) containing protease and phosphatase inhibitors. Lysates were incubated overnight at 4 °C with anti-EZR antibody or control IgG, then with protein A/G agarose beads for 4 h. Beads were washed five times, and immunoprecipitated proteins were analyzed by Western blotting with anti-CD44 and anti-EZR antibodies.

### Statistical analysis

Data were collected from three independent experiments or at least five mice per group and are presented as means ± standard deviation (SD). Statistical significance was assessed using one-way analysis of variance (ANOVA), two-way ANOVA, or Student's t-test, as appropriate, with GraphPad Prism 9.0 software. Significance levels were defined as follows: *P < 0.05, **P < 0.01, ***P < 0.001, and ****P < 0.0001.

## Results

### Ezrin and EMP1 promote lung cancer cell survival and metastatic potential under shear stress

Previously, to identify the key genes that help lung cancer cells survive and metastasize in circulation, we performed RNA sequencing (RNA-seq) analysis of A549 cells under 0 h, suspension and shear stress conditions (Fig. 1A). Further investigation revealed that PRSS3 and PAR2 played a vital role in promoting the invasion and metastasis of circulating lung cancer cells 30. However, how a subset of CTCs survived under SS and then metastasized to other sites was not investigated in that study. To further identify the important genes that promote the survival and metastasis of CTCs, we first compared the RNA expression levels from the RNA-seq data between the cells under suspension conditions and SS treatment. Suspension group was used to rule out the impacts of detachment on CTCs. We then selected the following six genes with significant higher elevation levels in SS treatment group: keratin 6A (KRT6A), hyaluronan synthase 2 (HAS2), limb bud and heart development homolog (LBH), EZR and epithelial membrane protein 1 (EMP1). We measured the mRNA levels of these six candidate genes by quantitative polymerase chain reaction (qPCR) and the results showed that their mRNA levels were all significantly elevated after SS treatment compared with suspension group (Fig. 1B). To establish a controlled system for mechanistic inquiry, we employed the A549 cell line instead of more heterogeneous patient-derived CTCs. This approach provided a homogeneous and genetically tractable platform essential for generating stable gene-modulated clones and performing high-throughput shear stress assays 32. In order to achieve higher gene expression for subsequent gene knockout studies, A-SSP6 cells were used. For further validation, we examined the expression of these genes in the SS-resistant cell line A-SSP6, which was generated in our lab via treating the lung cancer cell line A549-C3 with SS for 6 rounds 29 (Fig. S1A). To mitigate the effects of long-term culture on genetic and epigenetic profiles 33, 34, both A549-C3 and A-SSP6 cells were revived from early-passage frozen stocks. The q-PCR results showed that compared with the parental A549-C3 cells, the expression of HAS2, LBH, EZR and EMP1 was markedly upregulated by 4- to 15-fold in A-SSP6 cells, while KRT6A expression increased by 95.8-fold (Fig.1C).

To verify the roles of these genes in the survival and metastatic potential of CTCs, we knocked down them in A-SSP6 cells using small interfering RNAs (siRNAs) and detected the cell viability after SS and migration ability (Fig. S1B). The 10-hour shear stress served as a stringent functional selection protocol to mimic the extended circulatory phase of metastasis. Since most CTCs die within 3 hours 35, this duration enriches for the resilient subclones that survive beyond this point—precisely the population with the highest metastatic potential. Thus, studying these survivors reveals molecular adaptations that confer a lethal advantage. After knockdown of EZR and EMP1, the survival rates of A-SSP6 cells following 10 hours of SS treatment were reduced from around 60% to 24.7% and 34.3%, respectively, whereas knockdown of KRT6A, HAS2 and LBH did not significantly affect cell survival (Fig. 1D, E). These results suggest that EZR and EMP1 may serve as important mediators of lung cancer cell survival in circulation. To assess the critical step of metastatic colonization beyond initial survival, we performed a colony formation assay to measure clonogenic potential of the cells. As a result, low expression of EZR and EMP1 led to a 50% decrease in the colony formation ability of A-SSP6 cells (Fig. 1F, G). Similarly, survival in circulation means little if the cell cannot exit the vasculature. The migration assay is a direct functional proxy for this essential extravasation step. The migration ability was also markedly impaired, reducing to 29.1% and 42.5% of control cells, respectively (Fig. 1H, I). Taken together, our data suggest that EZR and EMP1 contribute to both survival and migratory capacity, supporting their potential involvement in multiple steps of the metastatic process.

### Low expression of EZR and EMP1 impair the survival and migration ability of A-SSP6 cells *in vitro* and *in vivo*

To further validate the function of EZR and EMP1 in circulation, short hairpin-mediated RNAs (shRNAs) were designed to generate stable knockdown cell lines. Two shRNAs were designed to target each gene, and both qPCR and Western blot results verified efficient knockdown (Fig. 2A-C).

Consistent with the results in Fig. 1D-I, knocking down EZR and EMP1 with shRNAs markedly reduced the SS survival rate of A-SSP6 cells from nearly 80% to 30% (Fig. 2 D, E). The colony formation and migration ability in A-SSP6-shEZR and A-SSP6-shEMP1 were also significantly reduced (Fig. 2F-I), supporting the involvement of EZR and EMP1 in promoting the survival and migration of circulating lung cancer cells *in vitro*.

Next, to determine whether EZR and EMP1 were required for survival during circulation and subsequent colonization *in vivo*, we bypassed the initial steps of metastasis by directly injecting the A-SSP6 knockdown cells into the tail vein of NOD/SCID mice. The mice were sacrificed 4 h, 1, 2, 3 and 28 days post injection. The colony number in the lungs were counted, and the results showed that low expression of EZR and EMP1 impaired the metastatic ability of A-SSP6 cells by more than 80% after 28 days (Fig. 2J, K). Notably, a significant reduction in colony numbers was already observed 2-3 days post-injection, suggesting that EZR and EMP1 are crucial for immediate survival in circulation and early colonization. The timing aligns with the critical window when most CTCs are eliminated, consistent with a role in protecting cells from shear stress-induced apoptosis.

### High expression of EZR enhances the survival and migration ability of A549-C3 cells

To further investigate the potential roles of EZR and EMP1 in resisting SS damage, these two genes were overexpressed in A549-C3 cells (Fig. 3A-C). Then these cells were subjected to circulatory system for 10 h. As the results showed, overexpression of EZR and EMP1 increased the survival rate by around 20% compared with the empty vector group (Fig. 3D, E). The colony formation and migration ability were also enhanced. Specifically, the number of colonies nearly doubled in both overexpression cell lines (Fig. 3F, G), while the migrated cell number nearly tripled in the EZR-overexpressing group and doubled in the EMP1 overexpression group (Fig. 3H, I). For *in vivo* validation, tail vein injection of overexpression cells was conducted. The results showed that EZR overexpression significantly enhanced the number of lung colonies compared with the empty vector group, while no significant difference was observed in the EMP1-overexpressing group (Fig. 3J, K). Taken together, these results suggest that EZR overexpression promotes lung colonization in this model, whereas EMP1 overexpression, despite enhancing *in vitro* survival and migration, was insufficient to confer a similar advantage *in vivo* under the conditions tested.

To determine whether EZR and EMP1 act in parallel pathways or within a shared network to promote cell survival under shear stress conditions, we first examined their cross-regulation by qPCR. As shown in Fig. S2A, knockdown of EZR did not significantly alter EMP1 expression, and vice versa, suggesting no evident cross-regulation between these two genes at the transcriptional level.

To assess functional redundancy, we performed double knockdown of EZR and EMP1 in A-SSP6 cells (Fig. S2B) and subjected them to shear stress treatment (10 h). As shown in Fig. S2C-F, combined depletion of EZR and EMP1 did not produce a synergistic effect on cell survival or migration beyond that observed reduced effects with EZR knockdown alone (Fig. 2D, E, H, I), suggesting that, in this experimental setting, EMP1 does not substantially contribute to the core survival pathway beyond the effects mediated by EZR.

### EZR supports the metastasis of lung cancer cells in orthotopic models

Orthotopic models were used to mimic clinical lung cancer patients. This approach allows for a comprehensive assessment of EZR's impact on the entire metastatic cascade, from primary tumor growth to distant dissemination. To test the effects of EZR on the malignancy of circulating lung cancer cells in orthotopic models, EZR knockdown cells were injected into the lungs of NOD/SCID mice. In the control group, larger tumor area was observed compared with EZR knockdown group (Fig. 4A, B and S3A), as well as a much higher metastatic percentage (Fig. 4A, C). All the six mice in the control group had metastatic tumors in intestine, liver or brain, while in EZR knockdown group, only one mouse had metastatic tumors in the intestine and liver. The body weight did not change significantly between the two groups (Fig. 4D).

Subsequently, EZR overexpression cells were also subjected to the lung orthotopic model. The results showed that after 28 days, cells with high levels of EZR formed larger primary tumors in lungs (Fig. 4E, F and S3B), and also migrated more frequently into the intestine, liver and brain (Fig. 4E, G), with no significant change in the body weight observed (Fig. 4H). Together, these data suggest that EZR plays an important role in promoting the metastatic progression of lung cancer cells in this orthotopic model.

### EZR interacts with actin and CD44 at the cell membrane to protect CTCs in circulation and promote metastasis

After identifying the important role of EZR in helping lung cancer cells to resist SS damage in circulation, we conducted further research to investigate the underlying mechanisms. Ezrin acts as a membrane-actin linker to provide structural support and influence cell shape 36. To observe the changes of Ezrin and actin after circulation, we circulated EZR knockdown cells for 10 h and performed immunofluorescence staining. The results showed that knockdown of Ezrin led to abnormal distribution of actin, indicating altered cell shape after SS treatment in both A549-shEZR and H1975-shEZR cells (Fig. 5A). The percentage of cells with abnormal cell shape increased from 3.4% to 47.8% in the A549-shEZR cells which is 14.1-fold increment, while in H1975-shEZR cells, the percentage was increased from 6.7% to 56.9%, which is 8.5-fold increment (Fig. 5B). These observations suggest that Ezrin contributes to protecting CTCs from SS damage through interacting with actin to support cell structure and maintain normal cell shape.

Considering that Ezrin needs to be activated by phosphorylation to function as a cytoskeletal organization modulator, we used Western blotting to examine the activation of Ezrin after SS. The results showed that the protein levels of both EZR and phosphorylated Ezrin (p-EZR) were significantly upregulated, with p-EZR increasing to 14.8- and 6.9-fold in circulated A549 and H1975 lung cancer cells (Fig. 5C). In suspension state, the expression of p-EZR was also upregulated, but it was significantly increased in SS group (Fig. S4A). Since it is reported that CD44 can interact with EZR to support the migration of cancer cells, as CO-IP assay showed their interaction 37. we also checked the expression of CD44 and found that CD44 was also highly upregulated under SS conditions (Fig. 5C).

To further investigate the spatial distribution and potential interactions among Ezrin, actin, and CD44 during circulation, we performed immunofluorescence staining. In adherent cells, Ezrin localized mainly in the cytoplasm, and the level of p-EZR was low. While after SS treatment for 10 h, the levels of both EZR and p-EZR were increased, and they tended to assemble at the cell membrane. Actin displayed clear co-localization with EZR and p-EZR, suggesting enhanced Ezrin-mediated anchoring of the actin cytoskeleton to the membrane, thereby supporting cell structure under SS conditions (Fig. 5D, E). CD44 also co-localized with EZR and p-EZR at the cell membrane after SS treatment (Fig. 5D, E), which suggests a potential interaction between EZR and CD44 in CTCs. Changes in protein levels and location were observed in the suspension group, but they were less pronounced than those in the SS group (Fig. S4B, C), suggesting that SS treatment may specifically enhance the regulation of EZR. Together, these findings suggest that under SS conditions, activated EZR, potentially working with CD44, contributes to reinforcing the actin cytoskeleton-membrane linkage to help protect CTCs and maintain their structural integrity.

Immunofluorescence analysis revealed that EZR and CD44 partially colocalized at the cell membrane under static conditions, and this colocalization was markedly enhanced upon exposure to shear stress treatment. To quantitatively assess this observation, we performed colocalization analysis using Pearson's correlation coefficient (PCC) on 50 individual cells per condition from three independent experiments. As shown in Fig. S5A, shear stress treatment significantly increased the PCC for over 3.5-fold between EZR and CD44 compared between SS-treated cells and control cells under attached conditions. These data provide quantitative evidence for a shear stress-enhanced association between EZR and CD44 at the cell membrane.

To directly validate the physical interaction between EZR and CD44 and its regulation by shear stress and ROS, we performed co-immunoprecipitation (Co-IP) assays. As shown in Fig. S5B, shear stress treatment significantly enhanced the interaction between endogenous EZR and CD44 compared to control cells under attached conditions. Importantly, this shear stress-induced interaction was markedly attenuated by pretreatment with the ROS scavenger PG, indicating that ROS production is required for promoting EZR-CD44 association under flow conditions. Furthermore, CD44 knockdown abrogated shear stress-induced Ezrin phosphorylation at Thr567 (Fig. S5C), suggesting that the EZR-CD44 interaction is functionally important for Ezrin activation in this context.

### CD44 plays a vital role in promoting cell survival in circulation and promoting metastasis

Considering the reported interaction between EZR and CD44, we wondered if upregulated CD44 could help lung cancer cell survive in circulation and form metastatic tumors. We knocked down CD44 in A-SSP6 cells and overexpressed CD44 in A549-C3 cells to verify its function under SS conditions. After 10 hours of circulation, CD44 knockdown significantly reduced the survival rate of A-SSP6 cells, while overexpressing CD44 improved the surviving ability of A549-C3 cells (Fig. 6A, B, E, F). As for cell migration, low expression of CD44 greatly impaired the migrated cell number by more than 70% (Fig. 6C, G), whereas increased CD44 enhanced the migration ability of A549-C3 cells to 3-fold compared with the empty vector group (Fig. 6D, H). Lung colony formation assays further supported the role of CD44 in promoting metastasis. Knocking down CD44 decreased the *in vivo* metastatic ability of A-SSP6 cells by more than 90% (Fig. 6I, J), and consistently, overexpression of CD44 led to a 4-fold increase in the lung colony numbers (Fig. 6K, L). Together, these results suggest that CD44 plays an important role in supporting the survival, migration and metastatic potential of circulating lung cancer cells.

To further validate the relationship between EZR and CD44, the levels of EZR and p-EZR were examined through Western blot analysis in A-SSP6-shCD44 and A549-C3-OE-CD44 cells. The results revealed that knocking down the expression of CD44 greatly reduced the phosphorylation of EZR, but did not affect the level of EZR. Similarly, increasing the expression of CD44 elevated the level of p-EZR by 4.7-fold without affecting the level of EZR (Fig. 6M, N). This suggests that the expression level of CD44 influences the activation of EZR.

To determine whether CD44 regulates Ezrin phosphorylation by influencing its membrane localization, we performed cell fractionation followed by Western blot analysis. As shown in Fig. S6, shear stress treatment induced a marked increase in p-EZR levels in the membrane, but not in the cytosol fraction of the cells, which is consistent with its activation and membrane recruitment. These data suggest that CD44 is important for the stable membrane association of phosphorylated Ezrin under shear stress conditions.

### Phosphorylation of EZR at Thr567 induced by ROS is essential for the pro-survival role of EZR in circulating lung cancer cells

Previously, we have found that SS induces reactive oxygen species (ROS) production in breast cancer cells 38. In order to know whether elevated EZR is correlated with ROS, we measured ROS levels following SS treatment and examined the relationship between ROS and EZR expression. The results showed that after SS treatment, more circulated lung cancer cells were stained positive with a ROS-detective dye, and the percentage of ROS positive cells reached to the highest level of 40.7% to 48.7% at 6 h in both A549 and H1975 cells (Fig. 7A, B). When antioxidants of propyl gallate (PG) and N-acetyl cysteine (NAC) were added in the circulatory system, the production of ROS was restricted, and the upregulation of CD44 and the phosphorylation of EZR were also reduced (Fig. 7C). More importantly, treating A549 and H1975 cells with hydrogen peroxide which can produce ROS, markedly increased the protein levels of CD44 and p-EZR (Fig. 7D). These findings suggest that CD44 expression and EZR activation are upregulated in response to SS-induced ROS production.

To further validate the importance of SS treatment on increasing EZR phosphorylation, p-EZR inhibitor NSC668394 was used to treat A549 and H1975 cells during circulation. The Western blotting results showed that this inhibitor greatly prevented the big elevation of phosphorylated EZR after SS treatment (Fig. 7E). Cell viability after SS treatment was measured and the results showed that inhibiting EZR phosphorylation reduced the SS-survival rates of both A549 and H1975 cells (Fig. 7F, G). Furthermore, the phosphorylation of EZR mainly occurs at Thr567, which is responsible for the activation of EZR and subsequent mediation of cytoskeleton-membrane interactions 36. To verify the functional role of phosphorylation at this site, we overexpressed EZR with a phospho-null mutation which changed threonine (T) to alanine (A) at Thr567 in A549 and H1975 cells. The Western blotting results showed that the levels of EZR were slightly reduced, but the phosphorylation of EZR was completely prevented in T567A cells (Fig. 7H). Then these constructed cells were subjected to circulatory treatment, and the results showed that overexpression of wild type (WT) EZR enhanced the survival rate of A549 and H1975 cells, while overexpressing none-phosphorylated EZR with the T567A mutation failed to increase the viability of circulating lung cancer cells (Fig. 7I, J). Together, these results suggest that phosphorylation of EZR at Thr567, which is induced by the production of ROS upon SS treatment, is essential for the functional role of EZR to protect CTCs from SS damage.

To directly test whether CD44 and activated Ezrin function downstream of ROS to promote cell survival under shear stress, we performed functional rescue experiments. As shown in Fig. S7A and B, treatment with the ROS scavenger PG significantly reduced cell viability under shear stress (I could not see the reduction of viability from Fig. S7A, B, where is the date of SS without PG?). Notably, overexpression of CD44 partially but significantly rescued this viability impairment. More importantly, expression of a constitutively active Ezrin mutant (T567D) effectively restored cell viability to the levels that exceeded to untreated controls (Fig. S7C-E). These results provide evidence supporting that CD44 and activated Ezrin function downstream of ROS.

To directly establish whether shear stress and ROS induce Ezrin phosphorylation specifically at Thr567, we compared WT Ezrin and the phosphorylation-deficient T567A mutant upon shear stress treatment. As shown in Fig. S7F, shear stress markedly increased p-Ezrin levels in WT-expressing cells, but this increase was completely prevented in T567A-expressing cells, despite comparable total Ezrin expression. These findings indicate that shear stress treatment, via ROS, specifically targets Ezrin phosphorylation at Thr567, and that this residue is critical for Ezrin activation in response to hemodynamic stress.

To functionally validate whether RhoA/ROCK signaling acts as the upstream kinase module linking ROS to Ezrin activation, we treated cells with the specific RhoA inhibitor Rhosin under shear stress conditions. As shown in Fig. S7G, Rhosin significantly inhibited shear stress-induced Ezrin phosphorylation at Thr567, without affecting total Ezrin levels. This was accompanied by profound disruption of shear stress-adapted cell morphology (Fig. S7J, K) and a significant reduction in cell viability (Fig. S7H, I). Importantly, Rhosin treatment did not reduce shear stress-induced ROS levels (Fig. S7L, M), indicating that RhoA acts downstream of ROS. Together, these data suggest that RhoA signaling plays a functional role in shear stress-induced Ezrin phosphorylation, morphological adaptation, and cell survival, suggesting that RhoA serves as a key upstream kinase downstream of ROS in this mechanotransduction pathway.

To further investigate whether ubiquitin-proteasome degradation is involved in shear stress-induced Ezrin stabilization, we performed MG132 assay. As shown in Fig. S7N, MG132 significantly increased Ezrin levels in cells under static conditions, but SS treatment resulted in a much lesser elevation of Ezrin phosphorylation, suggesting that phosphorylated Ezrin may be subject to proteasomal-mediated degradation.

### EZR-mediated activation of PI3K-AKT-Bcl-2 and JNK/p38/NF-κB pathways enhances resistance to SS and metastatic potential in circulating lung cancer cells

In order to investigate the downstream pathways through which EZR protects CTCs to resist SS damage and promote metastasis, Western blot analyses were performed in EZR knockdown and overexpression cells. We first found that after knocking down of EZR, the levels of p-PI3K, p-AKT and Bcl-2 were significantly reduced (Fig. 8A), which is a vital pathway to support cell survival. When EZR was overexpressed, the PI3K/AKT/Bcl-2 pathway was upregulated accordingly (Fig. 8B). Interestingly, the level of p-AKT was increased by up to 6.9-fold after EZR overexpression. The AKT signaling pathway is a master regulator of cell survival. This pathway was reported by Li to play a significant role in forming cluster and promoting survival of CTCs in circulation 29. Our findings suggest that EZR may also contribute to this pathway to promote CTC survival. In addition, activation or phosphorylation of JNK, p38 and NF-κB was reduced following the knockdown of EZR and increased in EZR overexpression cells, revealing that their activation is regulated by levels and activation of EZR. The total level of JNK, p38 and NF-κB were not changed significantly after EZR and overexpression (Fig. S8A, B). These observations are consistent with a model in which EZR modulates JNK/p38 signaling to support cytoskeletal adaptation and survival under shear stress.

To determine the functional role of JNK and p38 in shear stress-exposed CTCs, we treated cells with specific inhibitors under shear stress conditions. As shown in Fig. S8C-F, inhibition of JNK (SP600125) or p38 (SB202190) significantly reduced cell viability, indicating that both pathways contribute to survival in this context.

To determine whether EZR upregulation is an early adaptive response rather than a late artifact, we performed a time-course analysis of cells exposed to shear stress. As shown in Fig. S8G, p-EZR increased as early as 2 h after shear stress exposure and continued to rise progressively. These results indicate that EZR upregulation is a rapid response initiated within the physiological circulation window of CTCs (1-3 h). The 10 h time point used in SS-related experiments in this study thus represents a more stringent selection condition, while the early induction at 2 h supports the physiological relevance of this pathway.

Then the clinical significance of EZR was investigated using Kaplan-Meier plotter. The clinical significance of EZR was underscored by its correlation with shorter overall survival (OS) and post-progression survival (PPS) of lung cancer patients (Fig. 8C). Furthermore, EZR expression increased with advancing pathological stages (Fig. 8D) and greater lymph node metastasis (Fig. 8E), consistent with its potential role in driving tumor progression and dissemination.

Through immunohistochemistry (IHC) staining of EZR and p-EZR in lung tumors and adjacent tissues from lung cancer patients, we further confirmed the positive correlation of EZR expression and activation with tumor progression, indicated by higher IHC scores in lung tumors (Fig. 8F, G). Collectively, these findings suggest the following model: when lung cancer cells enter the bloodstream, SS induces ROS production, which elevates CD44 and activates EZR. Subsequently, activated EZR translocates to the cell membrane, interacts with CD44, and reinforces the CD44-EZR-actin linkage to maintain normal cell shape and help CTCs resist SS damage. Downstream, p-EZR activates the PI3K/AKT/Bcl-2 pathway as well as JNK, p38 and NF-κB, which may collectively facilitate the survival and metastatic potential of circulating lung cancer cells (Fig. 8H).

## Discussion

Circulating tumor cells are a major source of cancer metastasis, yet only the small fraction that survives the circulatory environment succeeds in establishing distant colonies 39. Hemodynamic shear stress (SS) is a leading cause of CTC death 40. In this study, we describe a mechanosignaling pathway that may contribute to lung cancer CTC resistance to SS-induced damage, suggesting Ezrin as a key mediator of circulatory survival and metastatic potential.

Ezrin, a membrane-cytoskeleton linker protein, is frequently overexpressed in aggressive cancers and correlates with poor prognosis 41-45. While its role in metastasis is recognized, its specific function in protecting CTCs from mechanical stress has been unclear. Our findings indicate that Ezrin plays an important role in maintaining structural integrity under SS, as its knockdown led to rapid membrane-cytoskeleton disruption and cell death. This is supported by *in vivo* kinetics: Ezrin knockdown significantly reduced lung-colonizing cells as early as 24 hours after tail vein injection, a timeframe that is more consistent with acute circulatory failure than with a generalized proliferative defect.

Ezrin activation via phosphorylation at Thr567 is necessary for its function 36, 46, 47. Phosphorylated Ezrin (p-Ezrin) is linked to invasive phenotypes and poor survival in various cancers, including NSCLC 48-52. Here, we report that SS induces rapid Ezrin phosphorylation, and that pharmacological inhibition of this phosphorylation impairs CTC survival. The kinase responsible for this phosphorylation in our context appears to be ROCK, a downstream effector of RhoA. Our data indicate that RhoA expression is upregulated by SS, and specific RhoA inhibition prevents SS-induced Ezrin phosphorylation and survival. Importantly, SS triggers reactive oxygen species (ROS) production, and our data suggest that ROS acts as an upstream signal: antioxidant treatment markedly reduced Ezrin activation and subsequent survival. Taken together, these observations are consistent with a model in which SS initiates a signaling cascade where ROS production facilitates RhoA/ROCK-mediated Ezrin phosphorylation.

While JNK and p38 are often associated with pro-apoptotic signaling, their role is context-dependent. Under the transient shear stress conditions used in this study, pharmacological inhibition of either pathway significantly reduced CTC viability, suggesting that these pathways may contribute to cell survival in this particular context. This could involve integration with the concurrently activated PI3K/AKT pathway, potentially redirecting these stress kinases toward a protective outcome.

As a scaffold protein, phosphorylated Ezrin does not possess kinase activity. Instead, it functions as a necessary signal-organizing node. Inhibition of Ezrin phosphorylation abolishes the activation of both PI3K/AKT and JNK/p38 pathways under SS conditions, suggesting that p-Ezrin may serve as a critical scaffold enabling pro-survival signaling in this context. The precise molecular interactors linking p-Ezrin to these kinase cascades in this specific mechanobiological context remain to be fully mapped and represent an important direction for future work.

We further identify CD44 as a key co-factor in this pathway. SS treatment upregulates CD44, a cell adhesion molecule known to interact with Ezrin and enhance cancer cell invasiveness and stem-like properties 53-60. Our data suggest a functional hierarchy: ROS signaling acts upstream to promote CD44 upregulation and function. CD44, in turn, appears to serve as a membrane scaffold that is required for efficient membrane recruitment and subsequent phosphorylation of Ezrin. Knockdown of CD44 abolished SS-induced Ezrin phosphorylation and survival, even in the presence of elevated ROS. This points to CD44 as a signal-dependent amplifier that may convert a diffuse oxidative signal into a localized, productive cytoskeletal response. The physical and functional interaction between Ezrin and CD44 thus appears to be a key feature of the mechanoadaptive response.

To assess the specificity of this axis, we examined the parental cells (A549) from which the SS-resistant cells (A-SSP6) were selected. In those A549 cells, a short period of SS treatment (2 h) rapidly induced the phosphorylation of Ezrin, and Ezrin knockdown significantly impaired the survival of these cells. These observations suggest that the pathway identified in this study is not merely an artifact of long-term selection, and may represent a stress response that is amplified in resistant populations. The A-SSP6 line served as a useful experimental tool to enrich for and dissect this core survival module.

While EMP1 was co-identified with Ezrin in our transcriptomic screen, subsequent mechanistic dissection revealed that it operates in a distinct pathway. Double-knockdown experiments showed no synergy with Ezrin loss, and EMP1 overexpression failed to enhance metastasis *in vivo* in our experimental setting, despite improving *in vitro* migration and viability. These results suggest that, within the context of circulatory survival, Ezrin plays a more prominent role than EMP1.

The clinical relevance of Ezrin is supported by our analysis of patient data, which links high EZR expression with reduced progression-free and overall survival, advanced tumor stage, and greater nodal metastasis in lung cancer. The observation that SS-induced Ezrin upregulation *in vitro* mirrors its elevated expression in aggressive human tumors suggests a potential link between this mechanosensitive pathway and cancer progression in patients. We acknowledge that future validation in patient-derived CTCs or more heterogeneous *in vivo* models will be important to fully define the universality of this mechanism, and we note this as a direction for future investigation.

Several limitations of this study should be acknowledged. First, the 10-hour shear stress exposure used in our *in vitro* model exceeds the typical circulation half-life of most circulating tumor cells (CTCs), which is usually within 1-2 hours 61. However, as shown in our time-course experiments (Figure S8G), shear stress-induced Ezrin phosphorylation begins as early as 2 hours in A549 and H1975 cells, indicating that the key molecular events along the ROS-CD44-Ezrin axis are initiated within a physiologically relevant timeframe. The 10-hour time point was chosen to capture the maximal adaptive response and ensure robust biochemical detection. Nevertheless, we fully recognize that direct clinical evidence linking differential shear stress exposure durations to metastatic potential is currently lacking, and future studies using time-stamped CTC isolation or *in vivo* models will be important to address this question.

Second, while we have validated the ROS-CD44-Ezrin axis in multiple unselected lung cancer cell lines (A549 and H1975), the A-SSP6 line used in part of this study is a long-term shear stress-selected model that may exhibit a more pronounced Ezrin response compared to naïve CTCs. We acknowledge that A-SSP6 represents an extreme model of shear stress adaptation, which limits the direct translational generalizability of our findings. Ideally, validation in primary patient-derived CTCs would be the most clinically relevant approach. However, due to the extreme rarity of CTCs in patient blood (typically 1-10 cells per mL) 62, isolating sufficient viable CTCs for mechanistic assays such as shear stress exposure and western blotting is not feasible under current laboratory conditions. Therefore, we have used the A-SSP6 line as a valuable tool to dissect the molecular mechanism of shear stress adaptation, while emphasizing that future studies using CTC-derived cell lines, patient-derived xenograft models, or microfluidic-based live CTC expansion systems are necessary to confirm the broader applicability of our findings. Within the scope of this study, our conclusions are primarily mechanistic and should be interpreted with caution regarding clinical translation.

Finally, while our study delineates the downstream ROS-CD44-Ezrin effector module, the identity of the primary mechanosensor on the CTC surface remains an open question. Preliminary data from our lab suggest rapid calcium influx precedes ROS production, pointing to mechanosensitive ion channels as candidate initiators. A comprehensive exploration of this upstream event, however, extends beyond the scope of the present work and will be the focus of ongoing research.

In summary, our findings identify the ROS-CD44-Ezrin axis as an integrated mechanosignaling module that contributes to shear stress resistance in our experimental models. While the individual functions of Ezrin are well-established, the novelty of this work lies in revealing how these functions-structural reinforcement and pro-survival signaling-may be coordinately engaged within a specific mechanobiological context to overcome a key metastatic bottleneck. These observations suggest that this pathway may offer opportunities for therapeutic exploration; however, further validation in more clinically relevant models will be necessary to determine its translational potential.

## Supplementary Material

Supplementary figures and tables.

## Figures and Tables

**Figure 1:**
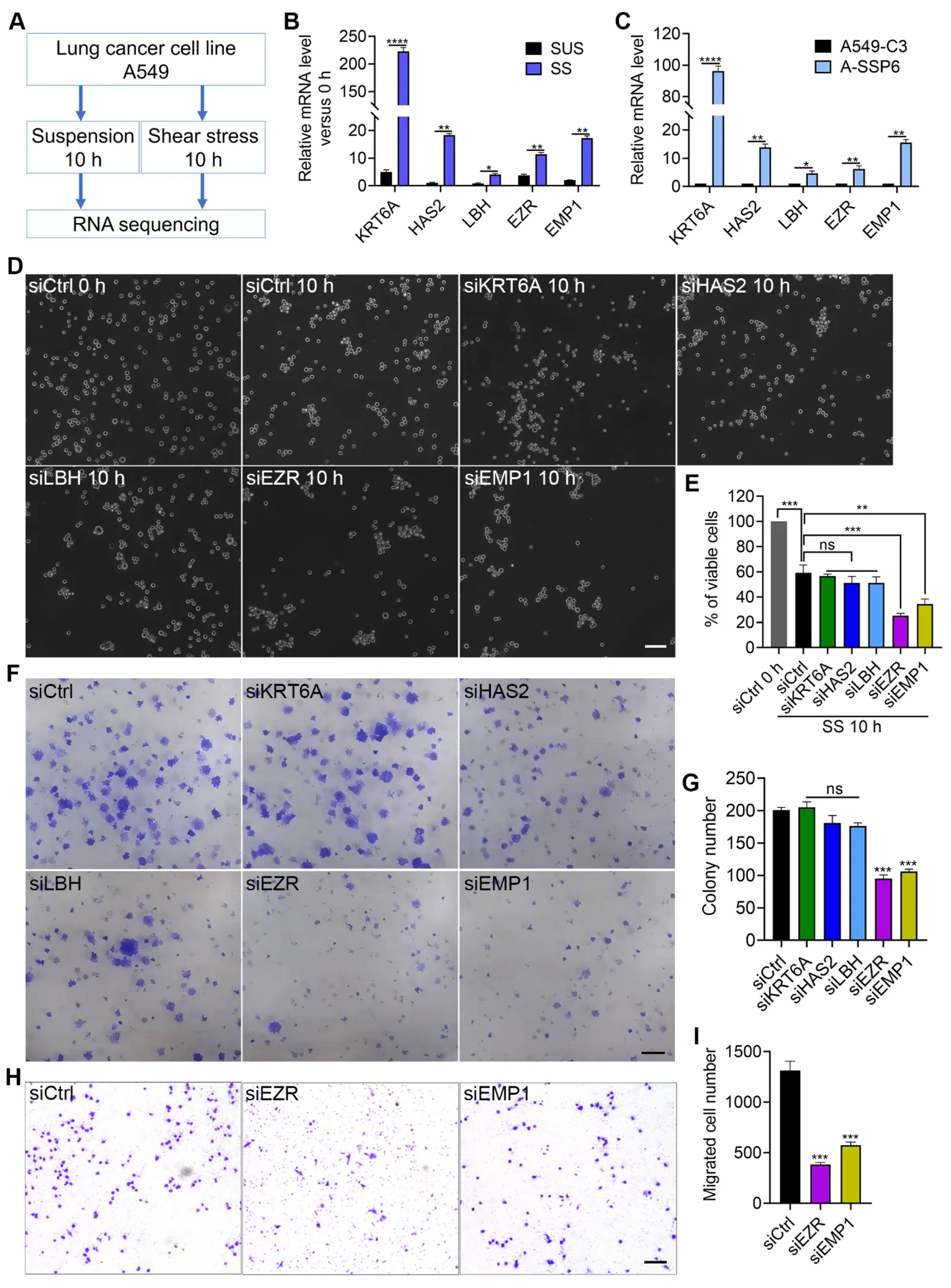
** Ezrin and EMP1 promote the survival and metastasis of lung cancer cells under circulatory conditions.** (A) Schematic picture showing the procedure of RNA sequencing. (B) qPCR results showing the relative mRNA levels of KRT6A, HAS2, LBH, EZR and EMP1 in A549 cells under suspension and SS conditions in comparison with those at 0 h. (C) qPCR results showing the relative mRNA levels of KRT6A, HAS2, LBH, EZR and EMP1 in A549-C3 and A-SSP6 cells. (D, E) Representative images and quantification results of the survival rates of cells under 10 h SS treatment after knocking down indicated genes. Scale bar: 100 μm. (F, G) Representative images and quantification results of the colony formation assay. One thousand knockdown cells were seeded in each well of 6-well plates and allowed to grow for 7 days. Scale bar: 2 mm. (H, I) Representative images and quantification results of the Transwell migration assay. Ten thousand cells were seeded and allowed to migrate for 18 h. Scale bar: 100 μm. The quantification results are the means ± SD from three independent experiments. Significant differences were determined by one-way ANOVA (E, G, I) and two-way ANOVA (B, C). *P < 0.05, **P < 0.01, ***P < 0.001, and ****P < 0.0001. ns, not significant.

**Figure 2:**
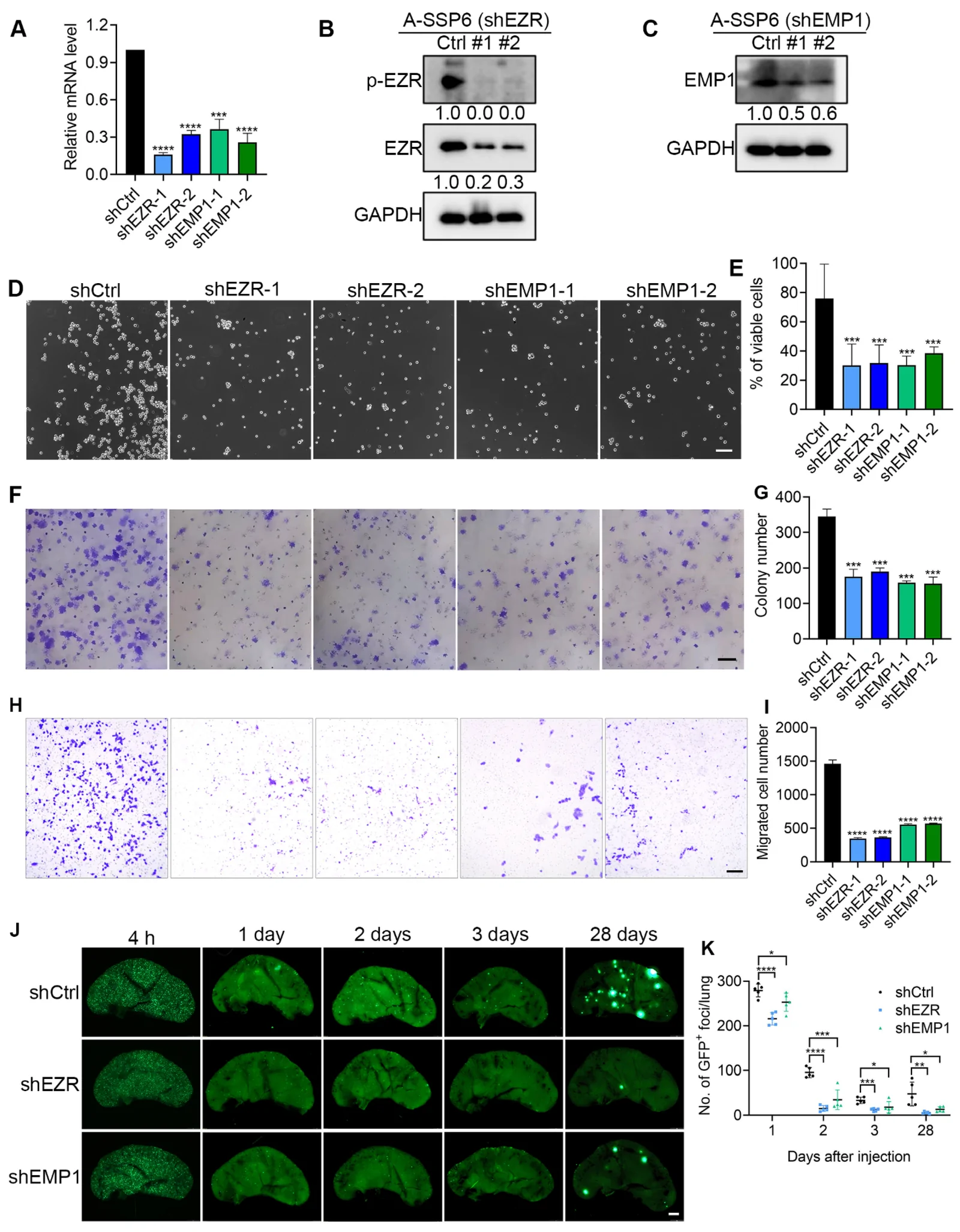
** Low expression of EZR and EMP1 impair the survival and migration ability of A-SSP6 cells *in vitro* and *in vivo*.** (A) qPCR results showing the relative mRNA levels of EZR and EMP1 after knockdown. (B, C) Western blot results showing the knockdown efficiency in A-SSP6 cells. (D, E) Representative images and quantification results of the survival rate of cells after knocking down EZR or EMP1. Scale bar: 100 μm. (F, G) Representative images and quantification results of the colony formation assay. Scale bar: 2 mm. (H, I) Representative images and quantification results of the Transwell migration assay. Scale bar: 100 μm. (J, K) Representative images and quantification results showing the lung colonies formed at different time points. Scale bar: 2 mm. The quantification results are the means ± SD from three independent experiments. Significant differences were determined by one-way ANOVA (A, E, G, I) and two-way ANOVA (K). *P < 0.05, **P < 0.01, ***P < 0.001, and ****P < 0.0001.

**Figure 3:**
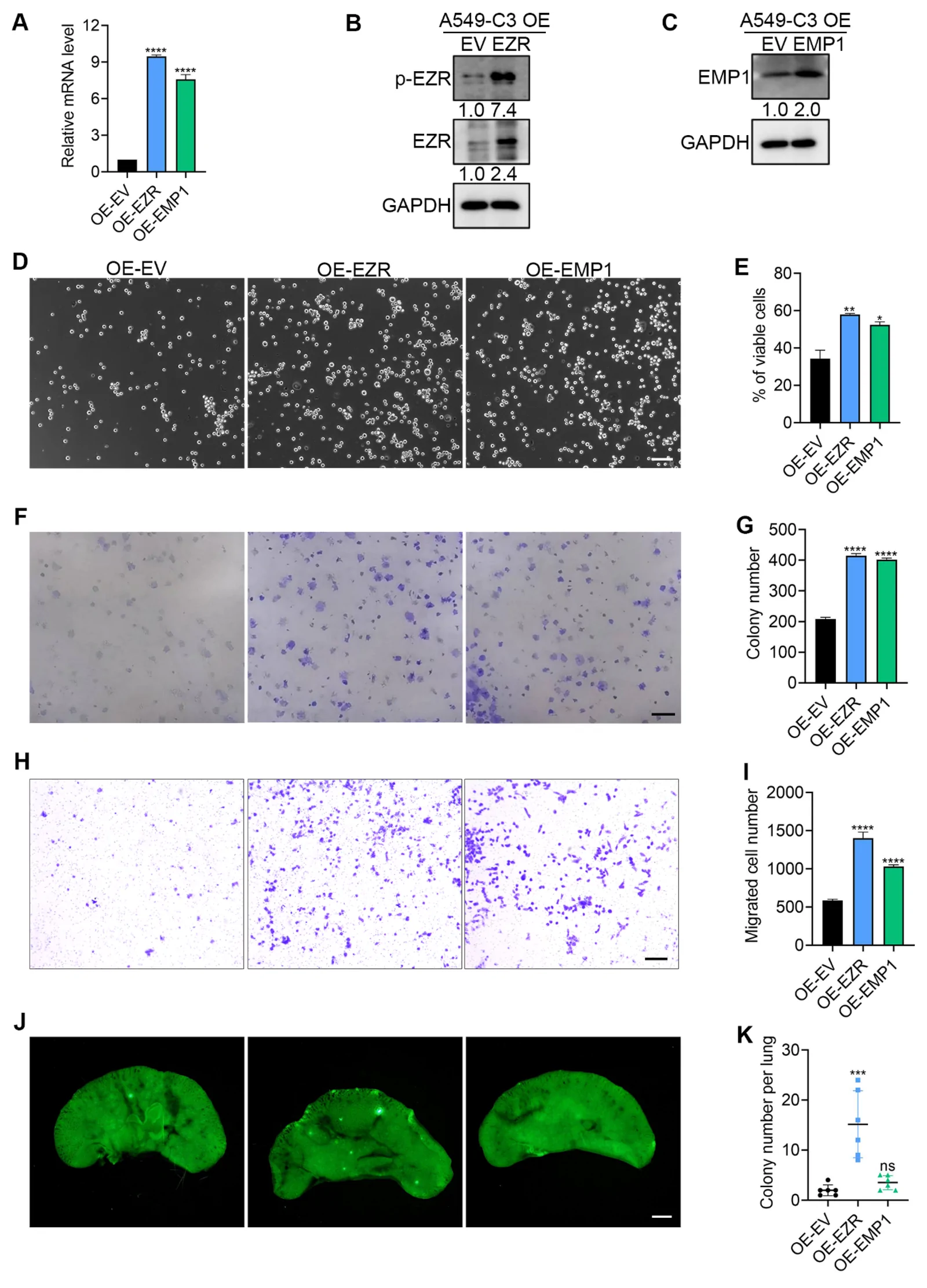
** High expression of EZR enhances the survival rate and migration ability of A549-C3 cells *in vitro* and *in vivo*.** (A) qPCR results showing the relative mRNA levels of EZR and EMP1 after overexpression. (B, C) Western blot results showing the efficiency of EZR and EMP1 overexpression in A549-C3 cells. (D, E) Representative images and quantification results of the survival rate of cells after overexpression. Scale bar: 100 μm. (F, G) Representative images and quantification results of the colony formation assay. Scale bar: 2 mm. (H, I) Representative images and quantification results of the Transwell migration assay. Scale bar: 100 μm. (J, K) Representative images and quantification results of the lung colony formation assay. Scale bar: 2 mm. The quantification results are the means ± SD from three independent experiments. Significant differences were determined by one-way ANOVA. *P < 0.05, **P < 0.01, ***P < 0.001, and ****P < 0.0001. ns, not significant.

**Figure 4:**
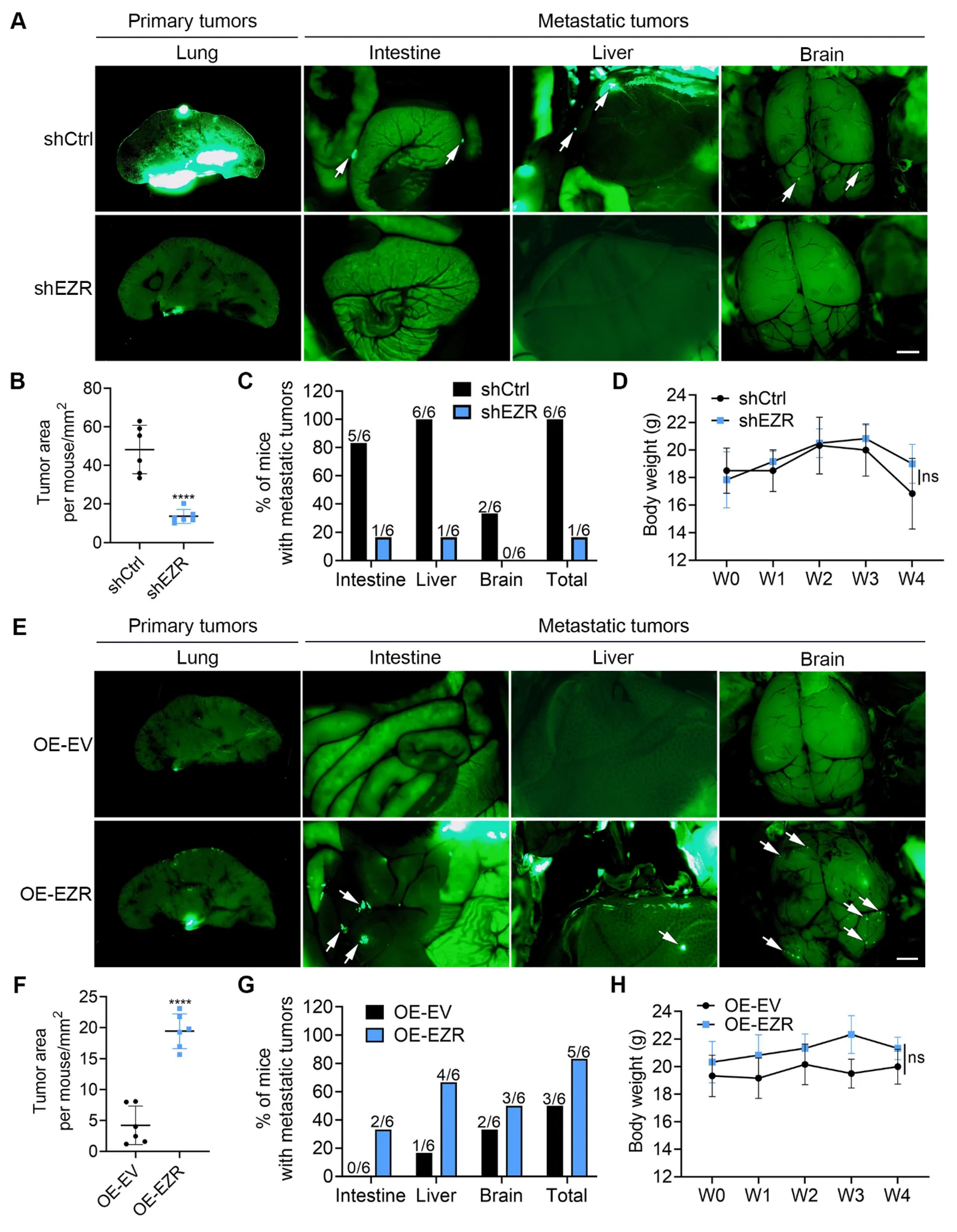
** EZR is important for the metastasis of lung cancer cells in orthotopic models.** (A, E) Representative fluorescent images of primary tumors in the lungs and metastatic tumors in the liver, intestine and brain (n = 6 mice per group). Four million cancer cells were orthotopically injected into the right lungs of each NOD/SCID mouse. Scale bar: 2 mm. White arrows indicate metastatic tumors. (B, F) The tumor area in all five lungs of each mouse was measured by ImageJ. (C, G) The percentage of mice with metastatic tumors in each group was calculated 4 weeks post injection. (D, H) The body weights of each mouse at 0, 1, 2, 3, and 4 weeks after orthotopic implantation were measured. The quantification results are the means ± SD from 6 mice. Significant differences were determined by t-test (B, F) and two-way ANOVA (D, H). ****P < 0.0001. ns, not significant.

**Figure 5:**
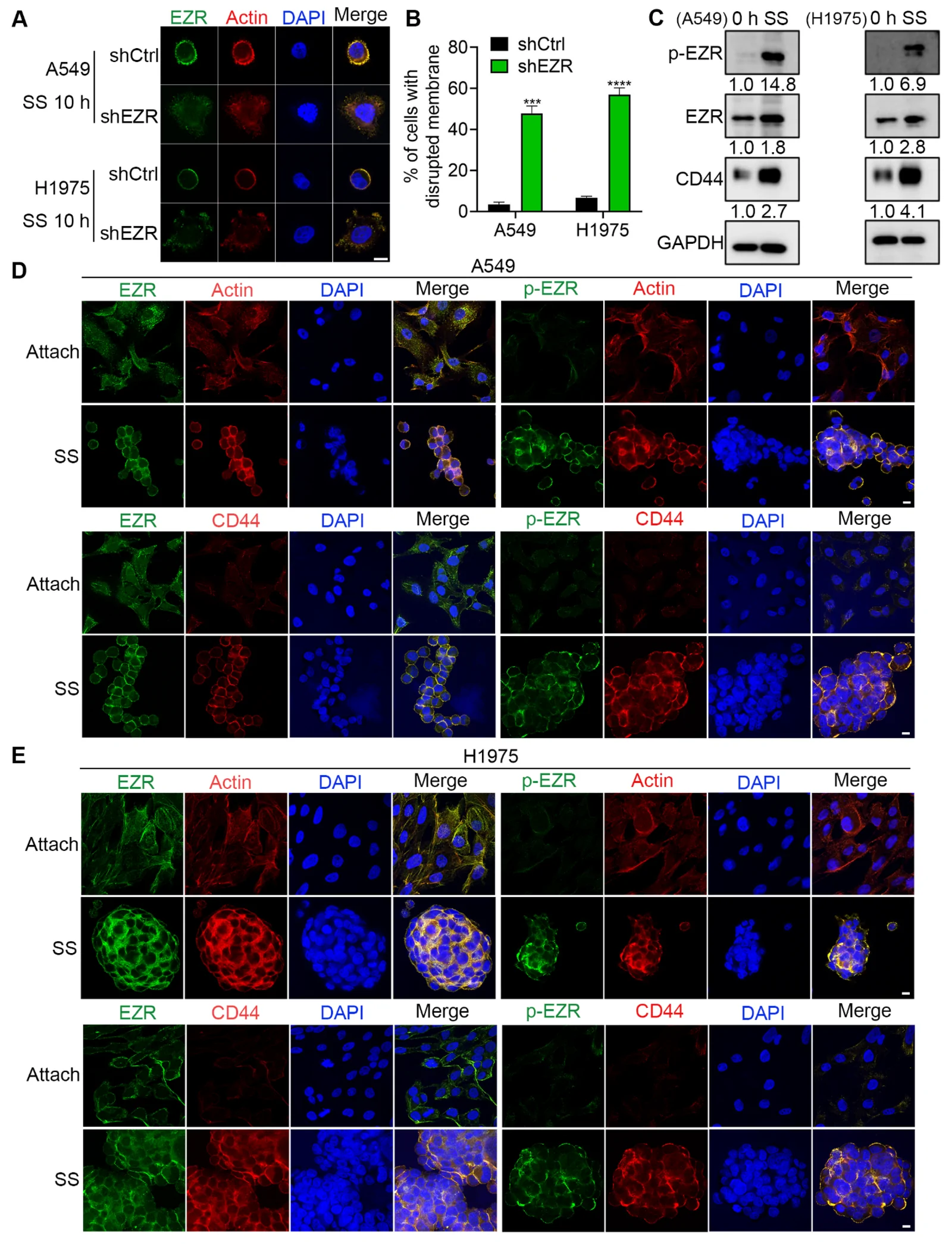
** Co-localization of EZR, p-EZR, CD44, and actin at the cell membrane is enhanced under SS.** (A) Representative immunofluorescence (IF) staining images showing the disrupted cell structure in A549-shEZR and H1975-shEZR cells after SS treatment. Scale bar: 5 μm. (B) Quantification result showing the percentage of cells with abnormal cell shape after circulation (n ≥ 100 cells). (C) Western blot results showing the protein levels of p-EZR, EZR and CD44 after SS treatment. (D, E) Representative IF staining images showing the distribution of EZR, p-EZR, actin and CD44 in A549 and H1975 cells. Scale bar: 10 μm. The quantification results are the means ± SD from three independent experiments. Significant differences were determined by two-way ANOVA. ***P < 0.001, and ****P < 0.0001.

**Figure 6:**
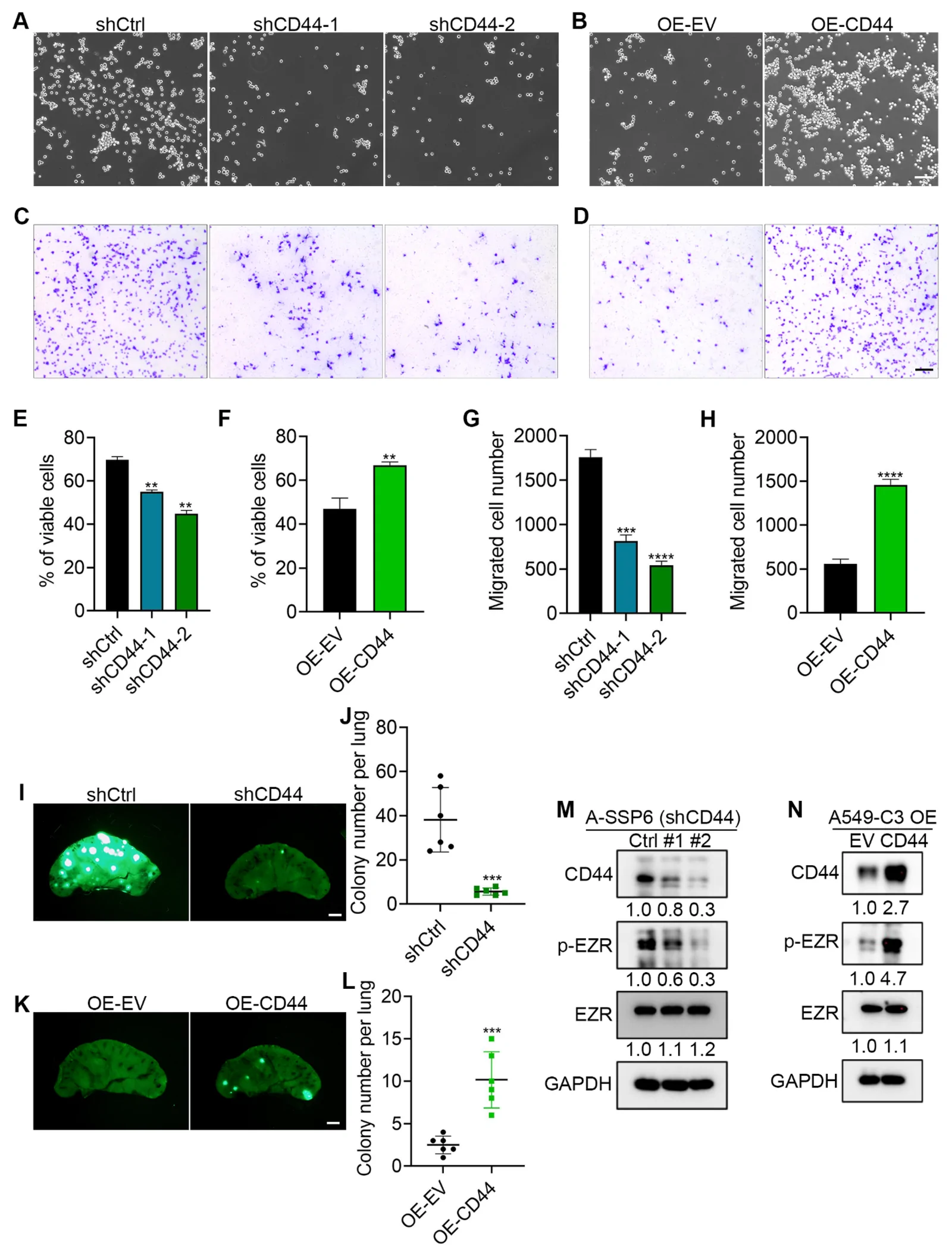
** CD44 is vital in protecting the cell survival in circulation and promoting metastasis.** (A, E) Representative images and quantification results of the cell survival rate after knocking down CD44. Scale bar: 100 μm. (B, F) Representative images and quantification results of the cell survival rate after overexpressing CD44. Scale bar: 100 μm. (C, G) Representative images and quantification results of the Transwell migration assay after knockdown of CD44. Scale bar: 100 μm. (D, H) Representative images and quantification results of the Transwell migration assay after overexpression of CD44. Scale bar: 100 μm. (I-L) Representative images (I, K) and quantification results (J, L) of the lung colony formation assay (n = 6 mice per group). Scale bar: 2 mm. (M, N) Western blot results showing the knockdown and overexpression efficiency of CD44, and the relative changes of EZR and p-ZER. The quantification results are the means ± SD from three independent experiments or more than five mice. Significant differences were determined by one-way ANOVA (E, G) and t-test (F, H, J, L). **P < 0.01, ***P < 0.001, and ****P < 0.0001.

**Figure 7:**
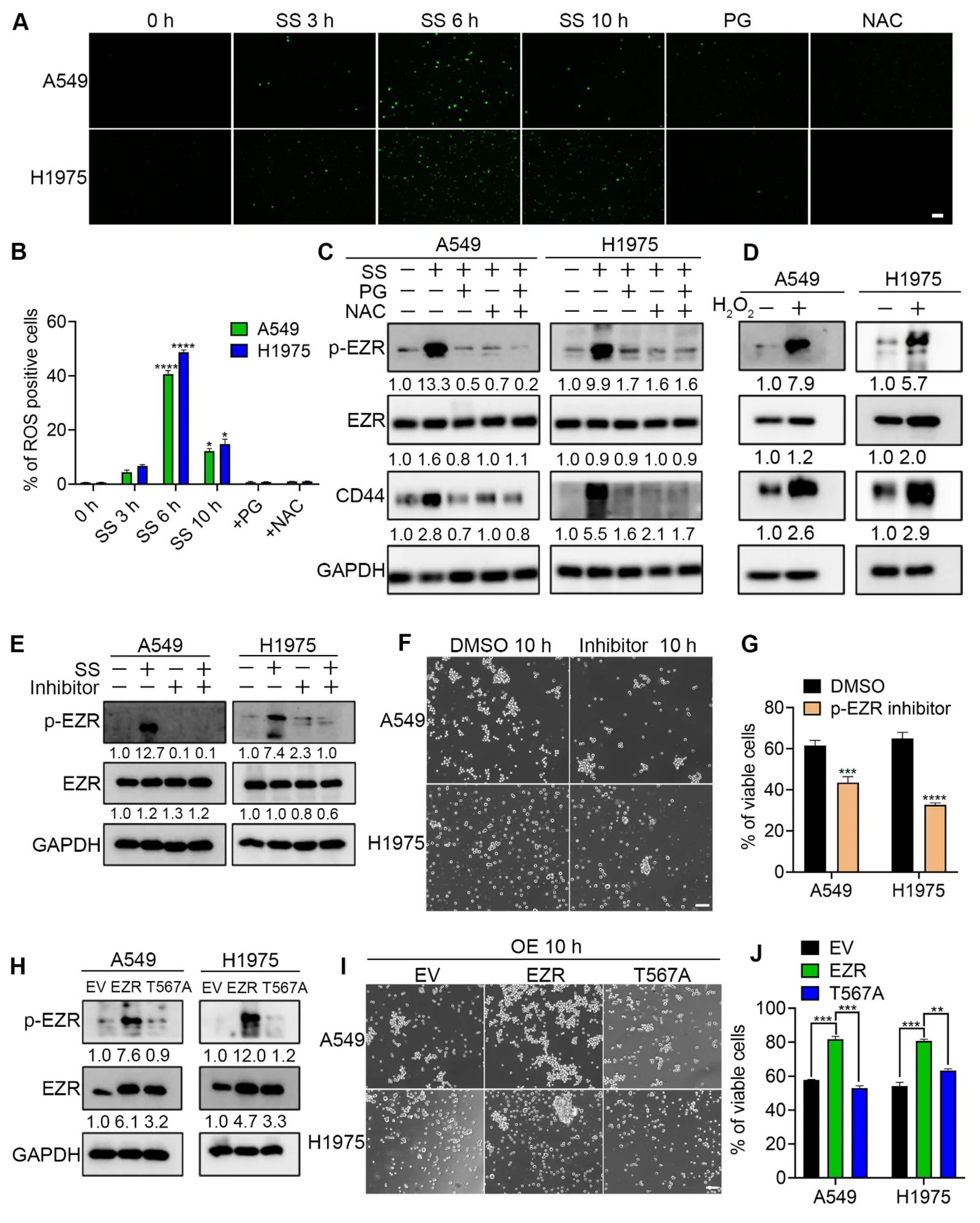
** Activation of EZR induced by ROS is important for the survival of lung cancer cells in circulation.** (A, B) Representative images and quantification results showing the ROS levels after SS treatment for different time points with or without PG and NAC. PG: 20 μM, NAC: 5 mM, Scale bar: 100 μm. (C) Western blot results showing the protein levels of CD44, EZR and p-EZR after SS treatment with or without PG and NAC. (D) Western blot results showing the protein levels of CD44, EZR and p-EZR after H_2_O_2_ treatment. (E) Western blot results showing the protein levels of EZR and p-EZR after using p-EZR inhibitor. (F, G) Representative images and quantification results showing the survival rate of cells after SS treatment with or without the addition of p-EZR inhibitor. Scale bar: 100 μm. (H) Western blot results showing the protein levels of EZR and p-EZR in cells with EZR or EZR (T567A) overexpression. (I, J) Representative images and quantification results showing the SS-survival rate of cells with EZR or EZR (T567A) overexpression. Scale bar: 100 μm. The quantification results are the means ± SD from three independent experiments. Significant differences were determined by two-way ANOVA. *P < 0.05, **P < 0.01, ***P < 0.001, and ****P < 0.0001.

**Figure 8:**
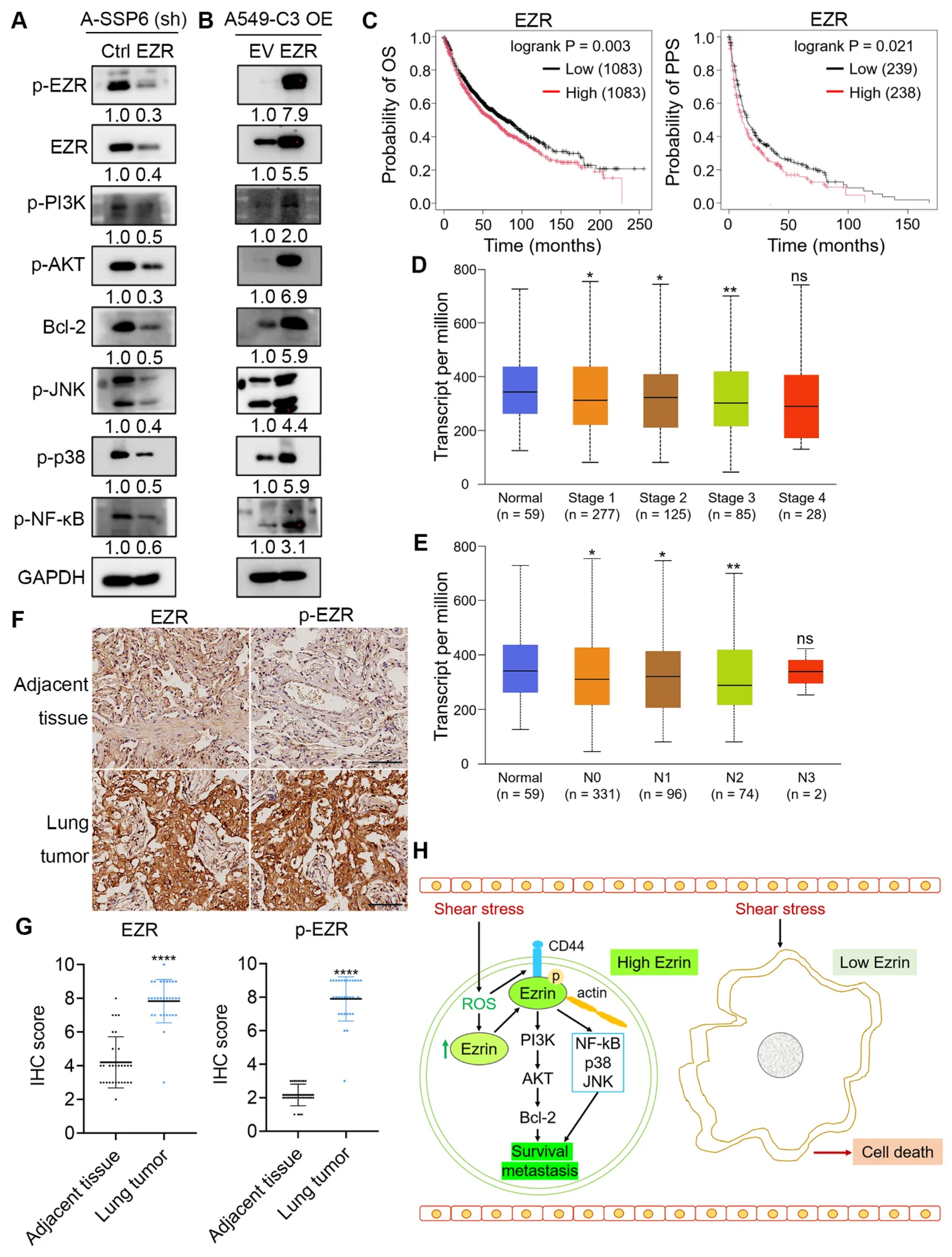
** EZR promotes survival and metastasis of circulating tumor cells by activating PI3K/AKT/Bcl-2, JNK, p38 and NF-κB pathways.** (A, B) Western blot results showing the protein levels of potential downstream molecules after knocking down or overexpressing EZR. (C) Kaplan-Meier plots of OS and PPS curves in lung cancer patients. (D) EZR expression in different stages of lung cancer patients. (E) EZR expression in lung cancer patients with different nodal metastasis. N0: No regional lymph node metastasis, N1: Metastases in 1 to 3 axillary lymph nodes, N2: Metastases in 4 to 9 axillary lymph nodes, N3: Metastases in 10 or more axillary lymph nodes. (F, G) Representative images and quantified IHC scores of EZR and p-EZR in adjacent tissues and lung tumors of NSCLC patients (n = 30). Scale bar: 100 μm. (H) Proposed signaling pathways. Significant differences were determined by Log-rank test (C) and Student's t-test (D, E, G). *P < 0.05, **P < 0.01, and ****P <0.0001. ns, not significant.

## Data Availability

Data generated in this study are available within the article and its supplementary data files and are available upon request from the corresponding author.

## References

[1] Schabath MB, Cote ML (2019). Cancer progress and priorities: lung cancer. Cancer Epidemiol Biomarkers Prev.

[2] Castro-Giner F, Aceto N (2020). Tracking cancer progression: from circulating tumor cells to metastasis. Genome Med.

[3] Gradilone A, Naso G, Raimondi C, Cortesi E, Gandini O, Vincenzi B (2011). Circulating tumor cells (CTCs) in metastatic breast cancer (MBC): prognosis, drug resistance and phenotypic characterization. Ann Oncol.

[4] O'Flaherty JD, Gray S, Richard D, Fennell D, O'Leary JJ, Blackhall FH (2012). Circulating tumour cells, their role in metastasis and their clinical utility in lung cancer. Lung Cancer.

[5] Hardingham JE, Grover P, Winter M, Hewett PJ, Price TJ, Thierry B (2015). Detection and clinical significance of circulating tumor cells in colorectal cancer-20 years of progress. Mol Med.

[6] Danila DC, Fleisher M, Scher HI (2011). Circulating tumor cells as biomarkers in prostate cancer. Clin Cancer Res.

[7] Gupta GP, Massagué J (2006). Cancer metastasis: building a framework. Cell.

[8] Massagué J, Obenauf AC (2016). Metastatic colonization by circulating tumour cells. Nature.

[9] Chambers AF, Groom AC, MacDonald IC (2002). Dissemination and growth of cancer cells in metastatic sites. Nat Rev Cancer.

[10] Fidler IJ, Kripke ML (1977). Metastasis results from preexisting variant cells within a malignant tumor. Science.

[11] Liotta LA, Kohn EC (2001). The microenvironment of the tumour-host interface. Nature.

[12] Mehlen P, Puisieux A (2006). Metastasis: a question of life or death. Nat Rev Cancer.

[13] Hong Y, Fang F, Zhang Q (2016). Circulating tumor cell clusters: What we know and what we expect (Review). Int J Oncol.

[14] Wirtz D, Konstantopoulos K, Searson PC (2011). The physics of cancer: the role of physical interactions and mechanical forces in metastasis. Nat Rev Cancer.

[15] Turitto VT (1982). Blood viscosity, mass transport, and thrombogenesis. Prog Hemost Thromb.

[16] Huang Q, Hu X, He W, Zhao Y, Hao S, Wu Q (2018). Fluid shear stress and tumor metastasis. Am J Cancer Res.

[17] Ortiz-Otero N, Marshall JR, Lash B, King MR (2020). Chemotherapy-induced release of circulating tumor cells into the bloodstream in collective migration units with cancer-associated fibroblasts in metastatic cancer patients. BMC Cancer.

[18] Lee HJ, Diaz MF, Price KM, Ozuna JA, Zhang S, Sevick-Muraca EM (2017). Fluid shear stress activates YAP1 to promote cancer cell motility. Nat Commun.

[19] Regmi S, Fu A, Luo KQ (2017). High shear stresses under exercise condition destroy circulating tumor cells in a microfluidic system. Sci Rep.

[20] Xin Y, Li K, Yang M, Tan Y (2020). Fluid shear stress induces EMT of circulating tumor cells via JNK signaling in favor of their survival during hematogenous dissemination. Int J Mol Sci.

[21] Thamilselvan V, Patel A, van der Voort van Zyp J, Basson MD (2004). Colon cancer cell adhesion in response to Src kinase activation and actin-cytoskeleton by non-laminar shear stress. J Cell Biochem.

[22] Li S, Chen Y, Zhang Y, Jiang X, Jiang Y, Qin X (2018). Shear stress promotes anoikis resistance of cancer cells via caveolin-1-dependent extrinsic and intrinsic apoptotic pathways. J Cell Physiol.

[23] Mitchell MJ, King MR (2013). Physical biology in cancer. 3. The role of cell glycocalyx in vascular transport of circulating tumor cells. Am J Physiol Cell Physiol.

[24] Lee JY, Chung J, Kim KH, An SH, Kim M, Park J (2018). Fluid shear stress regulates the expression of Lectin-like oxidized low density lipoprotein receptor-1 via KLF2-AP-1 pathway depending on its intensity and pattern in endothelial cells. Atherosclerosis.

[25] Barnes JM, Nauseef JT, Henry MD (2012). Resistance to fluid shear stress is a conserved biophysical property of malignant cells. PLoS One.

[26] Mitchell MJ, Denais C, Chan MF, Wang Z, Lammerding J, King MR (2015). Lamin A/C deficiency reduces circulating tumor cell resistance to fluid shear stress. Am J Physiol Cell Physiol.

[27] Lawler K, O'Sullivan G, Long A, Kenny D (2009). Shear stress induces internalization of E-cadherin and invasiveness in metastatic oesophageal cancer cells by a Src-dependent pathway. Cancer Sci.

[28] Fu A, Ma S, Wei N, Tan BX, Tan EY, Luo KQ (2016). High expression of MnSOD promotes survival of circulating breast cancer cells and increases their resistance to doxorubicin. Oncotarget.

[29] Li K, Wu R, Zhou M, Tong H, Luo KQ (2021). Desmosomal proteins of DSC2 and PKP1 promote cancer cells survival and metastasis by increasing cluster formation in circulatory system. Sci Adv.

[30] Zhou M, Li K, Luo KQ (2023). Shear stress drives the cleavage activation of protease-activated receptor 2 by PRSS3/Mesotrypsin to promote invasion and metastasis of circulating lung cancer cells. Adv Sci.

[31] Choudhury KR, Yagle KJ, Swanson PE, Krohn KA, Rajendran JG (2010). A robust automated measure of average antibody staining in immunohistochemistry images. J Histochem Cytochem.

[32] Domcke S, Sinha R, Levine DA, Sander C, Schultz N (2013). Evaluating cell lines as tumour models by comparison of genomic profiles. Nat Commun.

[33] Ben-David U, Siranosian B, Ha G, Tang H, Oren Y, Hinohara K (2018). Genetic and transcriptional evolution alters cancer cell line drug response. Nature.

[34] Lorsch JR, Collins FS, Lippincott-Schwartz J (2014). Fixing problems with cell lines. Science.

[35] Meng S, Tripathy D, Frenkel EP, Shete S, Naftalis EZ, Huth JF (2004). Circulating tumor cells in patients with breast cancer dormancy. Clin Cancer Res.

[36] Bretscher A, Reczek D, Berryman M (1997). Ezrin: a protein requiring conformational activation to link microfilaments to the plasma membrane in the assembly of cell surface structures. J Cell Sci.

[37] Tsukita S, Oishi K, Sato N, Sagara J, Kawai A, Tsukita S (1994). ERM family members as molecular linkers between the cell surface glycoprotein CD44 and actin-based cytoskeletons. J Cell Biol.

[38] Ma S, Fu A, Chiew GG, Luo KQ (2016). Hemodynamic shear stress stimulates migration and extravasation of tumor cells by elevating cellular oxidative level. Cancer Lett.

[39] Fidler IJ (2003). The pathogenesis of cancer metastasis: the 'seed and soil' hypothesis revisited. Nat Rev Cancer.

[40] Hope JM, Bersi MR, Dombroski JA, Clinch AB, Pereles RS, Merryman WD (2021). Circulating prostate cancer cells have differential resistance to fluid shear stress-induced cell death. J Cell Sci.

[41] Zhang X, Flores LR, Keeling MC, Sliogeryte K, Gavara N (2020). Ezrin phosphorylation at T567 modulates cell migration, mechanical properties, and cytoskeletal organization. Int J Mol Sci.

[42] Meng Y, Lu Z, Yu S, Zhang Q, Ma Y, Chen J (2010). Ezrin promotes invasion and metastasis of pancreatic cancer cells. J Transl Med.

[43] Li L, Wang YY, Zhao ZS, Ma J (2011). Ezrin is associated with gastric cancer progression and prognosis. Pathol Oncol Res.

[44] Bruce B, Khanna G, Ren L, Landberg G, Jirström K, Powell C (2007). Expression of the cytoskeleton linker protein Ezrin in human cancers. Clin Exp Metastasis.

[45] Li J, Wei K, Yu H, Jin D, Wang G, Yu B (2015). Prognostic value of Ezrin in various cancers: A systematic review and updated meta-analysis. Sci Rep.

[46] Chen Y, Wang D, Guo Z, Zhao J, Wu B, Deng H (2011). Rho kinase phosphorylation promotes Ezrin-mediated metastasis in hepatocellular carcinoma. Cancer Res.

[47] Gautreau A, Louvard D, Arpin M (2000). Morphogenic effects of Ezrin require a phosphorylation-induced transition from oligomers to monomers at the plasma membrane. J Cell Biol.

[48] Ren L, Khanna C (2014). Role of Ezrin in osteosarcoma metastasis. Adv Exp Med Biol.

[49] Ren L, Hong SH, Chen QR, Briggs J, Cassavaugh J, Srinivasan S (2011). Dysregulation of Ezrin phosphorylation prevents metastasis and alters cellular metabolism in osteosarcoma. Cancer Res.

[50] Ren L, Hong SH, Cassavaugh J, Osborne T, Chou AJ, Kim SY (2008). The actin-cytoskeleton linker protein Ezrin is regulated during osteosarcoma metastasis by PKC. Oncogene.

[51] Saygıdeğer-Kont Y, Minas TZ, Jones H, Hour S, Çelik H, Temel I (2016). Ezrin enhances EGFR signaling and modulates erlotinib sensitivity in non-small cell lung cancer cells. Neoplasia.

[52] Li Q, Gao H, Xu H, Wang X, Pan Y, Hao F (2012). Expression of Ezrin correlates with malignant phenotype of lung cancer, and in vitro knockdown of Ezrin reverses the aggressive biological behavior of lung cancer cells. Tumor Biol.

[53] Martin TA, Harrison G, Mansel RE, Jiang WG (2003). The role of the CD44/Ezrin complex in cancer metastasis. Crit Rev Oncol Hematol.

[54] Legg JW, Lewis CA, Parsons M, Ng T, Isacke CM (2002). A novel PKC-regulated mechanism controls CD44-Ezrin association and directional cell motility. Nat Cell Biol.

[55] Ma L, Jiang T (2013). Clinical implications of Ezrin and CD44 co-expression in breast cancer. Oncol Rep.

[56] Harrison GM, Davies G, Martin TA, Jiang WG, Mason MD (2002). Distribution and expression of CD44 isoforms and Ezrin during prostate cancer-endothelium interaction. Int J Oncol.

[57] Chen C, Zhao S, Karnad A, Freeman JW (2018). The biology and role of CD44 in cancer progression: therapeutic implications. J Hematol Oncol.

[58] Bhattacharya R, Mitra T, Ray Chaudhuri S, Roy SS (2018). Mesenchymal splice isoform of CD44 (CD44s) promotes EMT/invasion and imparts stem-like properties to ovarian cancer cells. J Cell Biochem.

[59] Xu H, Tian Y, Yuan X, Liu Y, Wu H, Liu Q (2016). Enrichment of CD44 in basal-type breast cancer correlates with EMT, cancer stem cell gene profile, and prognosis. Onco Targets Ther.

[60] Cho SH, Park YS, Kim HJ, Kim CH, Lim SW, Huh JW (2012). CD44 enhances the epithelial-mesenchymal transition in association with colon cancer invasion. Int J Oncol.

[61] Moose DL, Krog BL, Kim TH, Zhao L, Williams-Perez S, Burke G (2020). Cancer cells resist mechanical destruction in circulation via RhoA/actomyosin-dependent mechano-adaptation. Cell Rep.

[62] Alvarez Cubero MJ, Lorente JA, Robles-Fernandez I, Rodriguez-Martinez A, Puche JL, Serrano MJ (2017). Circulating tumor cells: markers and methodologies for enrichment and detection. Methods Mol Biol.

