# Supplementary material for: Ezrin Functions as a Key Mechanosensitive Effector to Preserve Cell Structure and Promote Survival and Metastasis of Lung Cancer Cells during Circulation
Source: Int J Biol Sci. 2026 Sep 3;22(14):7887–907. doi: 10.7150/ijbs.127134 (PMC13618229; doi:10.7150/ijbs.127134)
Supplement: Supplementary file 1 [file ijbsv22p7887s1.pdf]

Supplementary Information

# Ezrin Functions as a Key Mechanosensitive Effector to Preserve Cell Structure and Promote Survival and Metastasis of Lung Cancer Cells during Circulation

Xiaoying Zhang[1#], Muya Zhou[1#], Koukou Li[1], Mingheng Yuan[1], Haibo Tong[1], Kathy Qian Luo[1,2✉]

1. Department of Biomedical Sciences, Faculty of Health Sciences, University of Macau, Taipa, Macao SAR, China.
2. Ministry of Education Frontiers Science Center for Precision Oncology, University of Macau.

# Xiaoying Zhang and Muya Zhou contributed equally to this work.

✉ Corresponding author: Kathy Qian Luo, Department of Biomedical Sciences, Faculty of Health Sciences, University of Macau, Taipa, Macao SAR, China. Tel: 853-8822-4233; Fax: 853-8822-2314; Email: kluo@um.edu.mo.

**Supplementary figures**

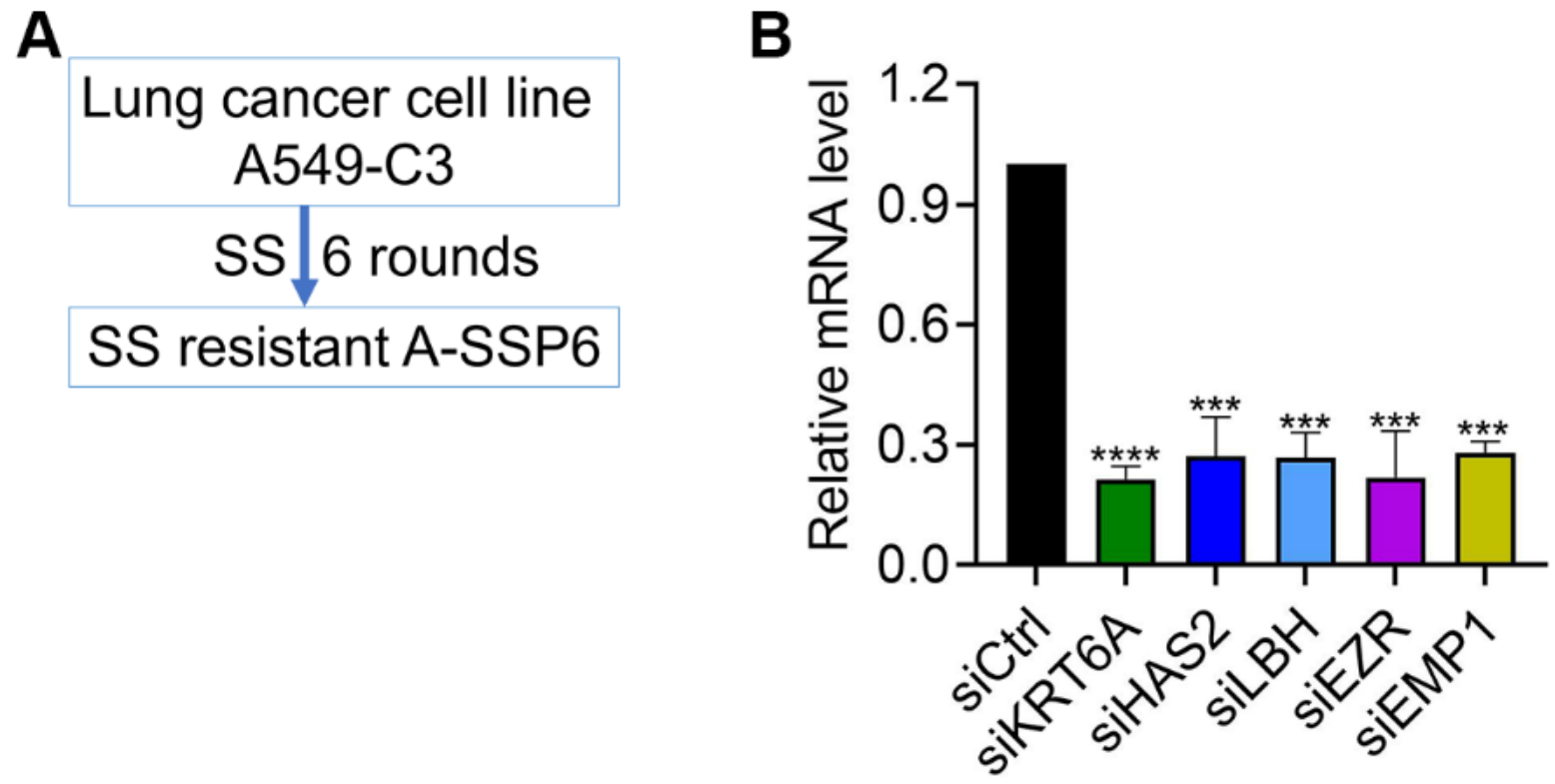


**Fig. S1.** (A) Schematic illustration of the derivation process for the shear stress-resistant cell line A-SSP6, generated through six cycles of circulatory selection from the parental A549-C3 lung adenocarcinoma cell line. (B) qPCR results showing the relative mRNA levels of KRT6A, HAS2, LBH, EZR and EMP1 after knockdown. The quantification results are the means ± SD from three independent experiments. Significant differences were determined by one-way ANOVA. ***$P < 0.001$, and ****$P < 0.0001$.

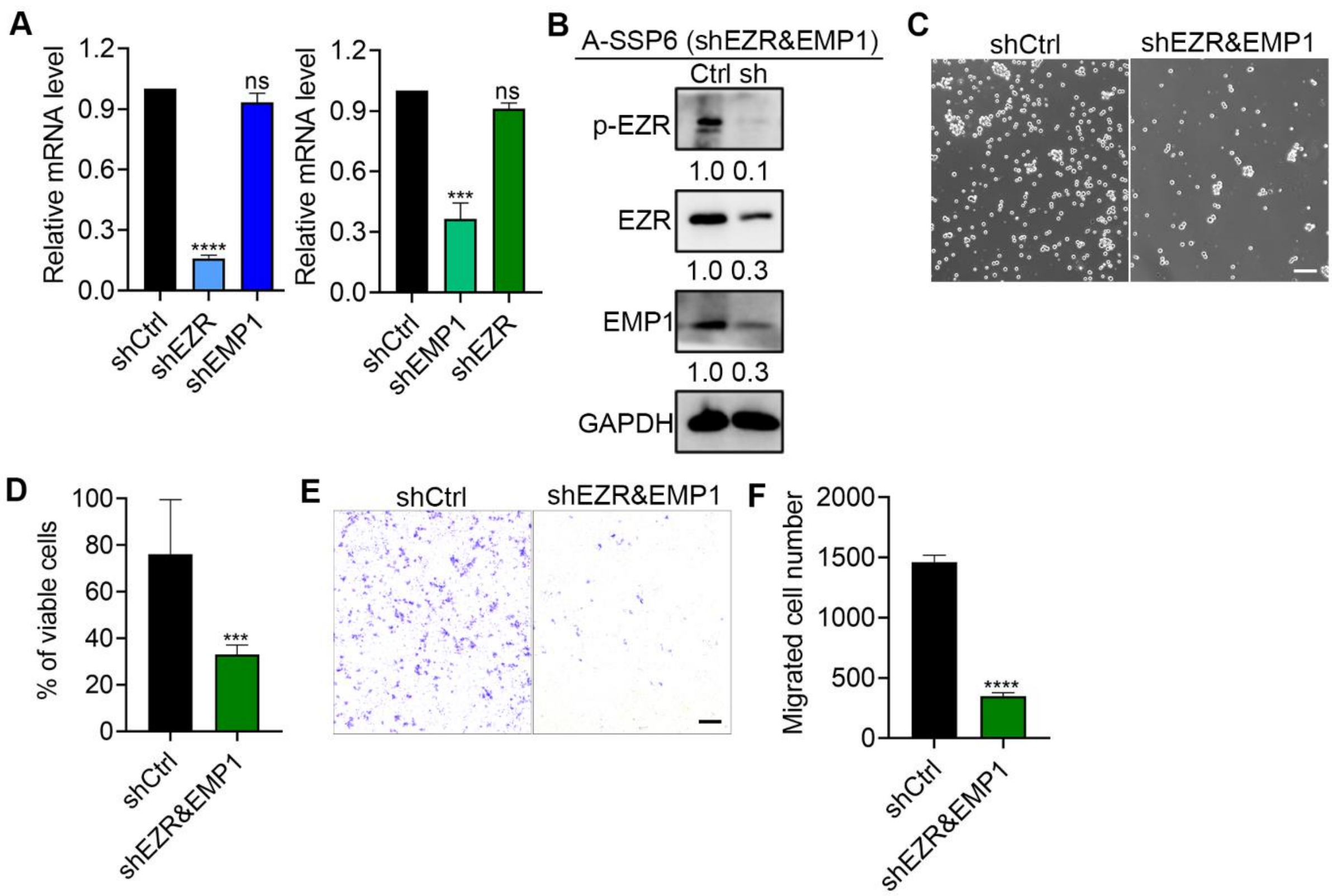


**Fig. S2.** (A) qPCR results showing the relative mRNA levels of EZR and EMP1 after EZR and EMP1 knockdown. (B) Western blot results showing the double knockdown efficiency in A-SSP6 cells. (C, D) Representative images and quantification results of the survival rate of cells after double knocking down of EZR and EMP1. Scale bar: 100 μm. (E, F) Representative images and quantification results of the Transwell migration assay after double knocking down

of EZR and EMP1. Scale bar: 100 μm. The quantification results are the means ± SD from three independent experiments. Significant differences were determined by one-way ANOVA. ***P < 0.001, and ****P < 0.0001. ns, not significant.

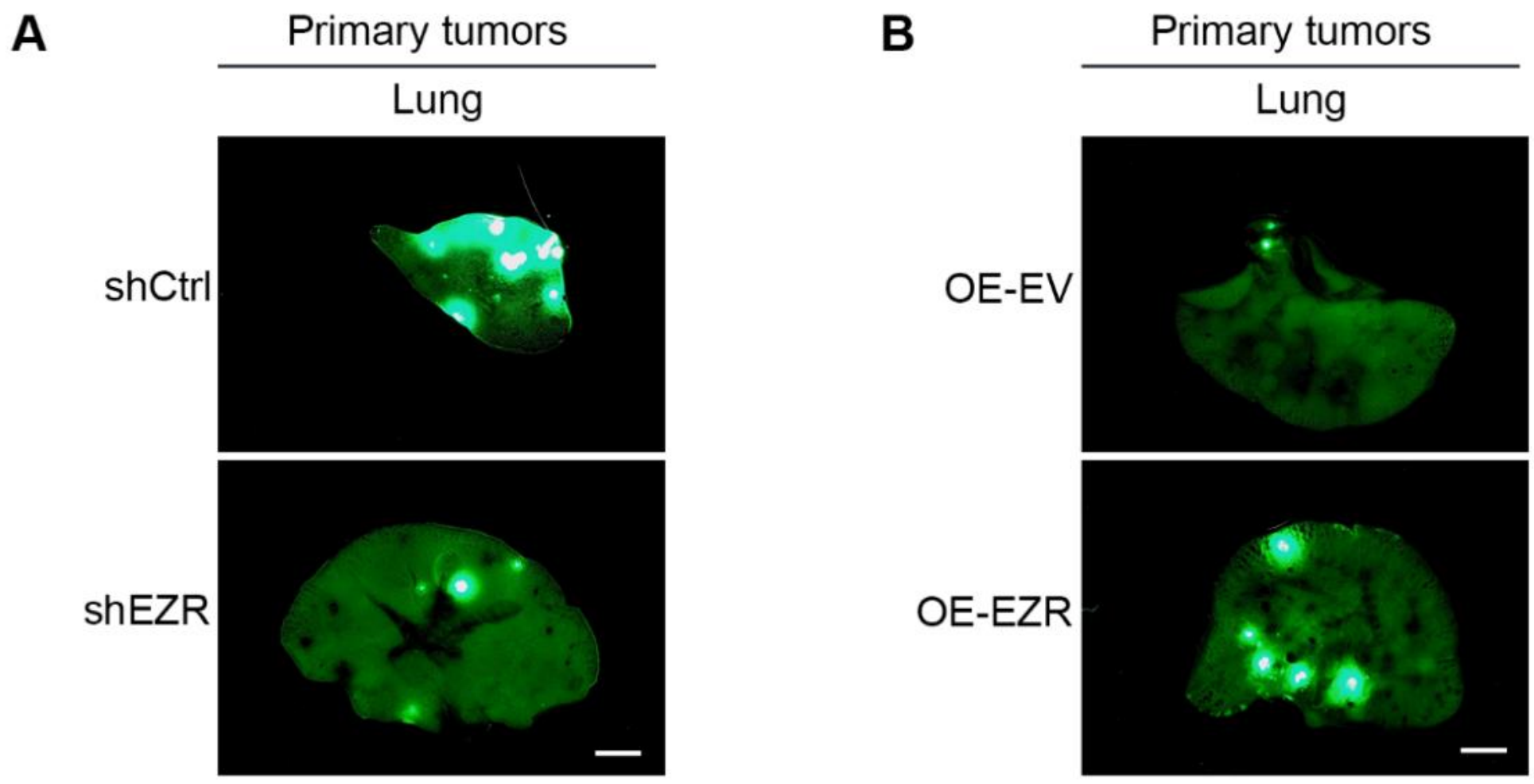


**Fig. S3.** (A, B) Representative images of other lung lobes in orthotopic models, showing additional primary tumor colonies. Scale bar: 2 mm.

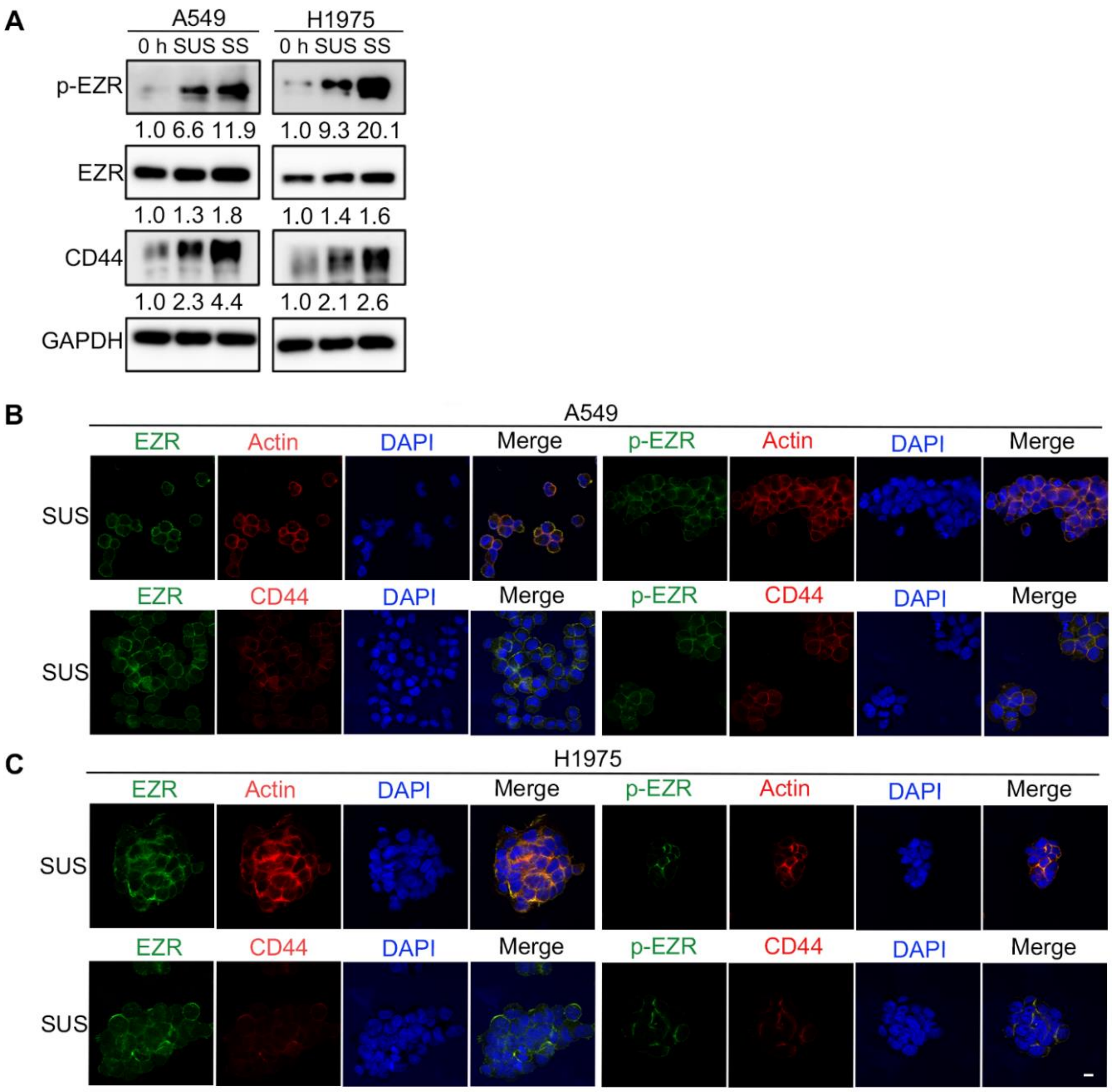


**Fig. S4.** (A) Western blot results showing the protein levels of p-EZR, EZR and CD44 after suspension and SS treatment. (B, C) Representative immunofluorescence (IF) staining images showing the distribution of EZR, p-EZR, actin and CD44 in A549 and H1975 cells under suspension conditions. Scale bar: 10 μm.

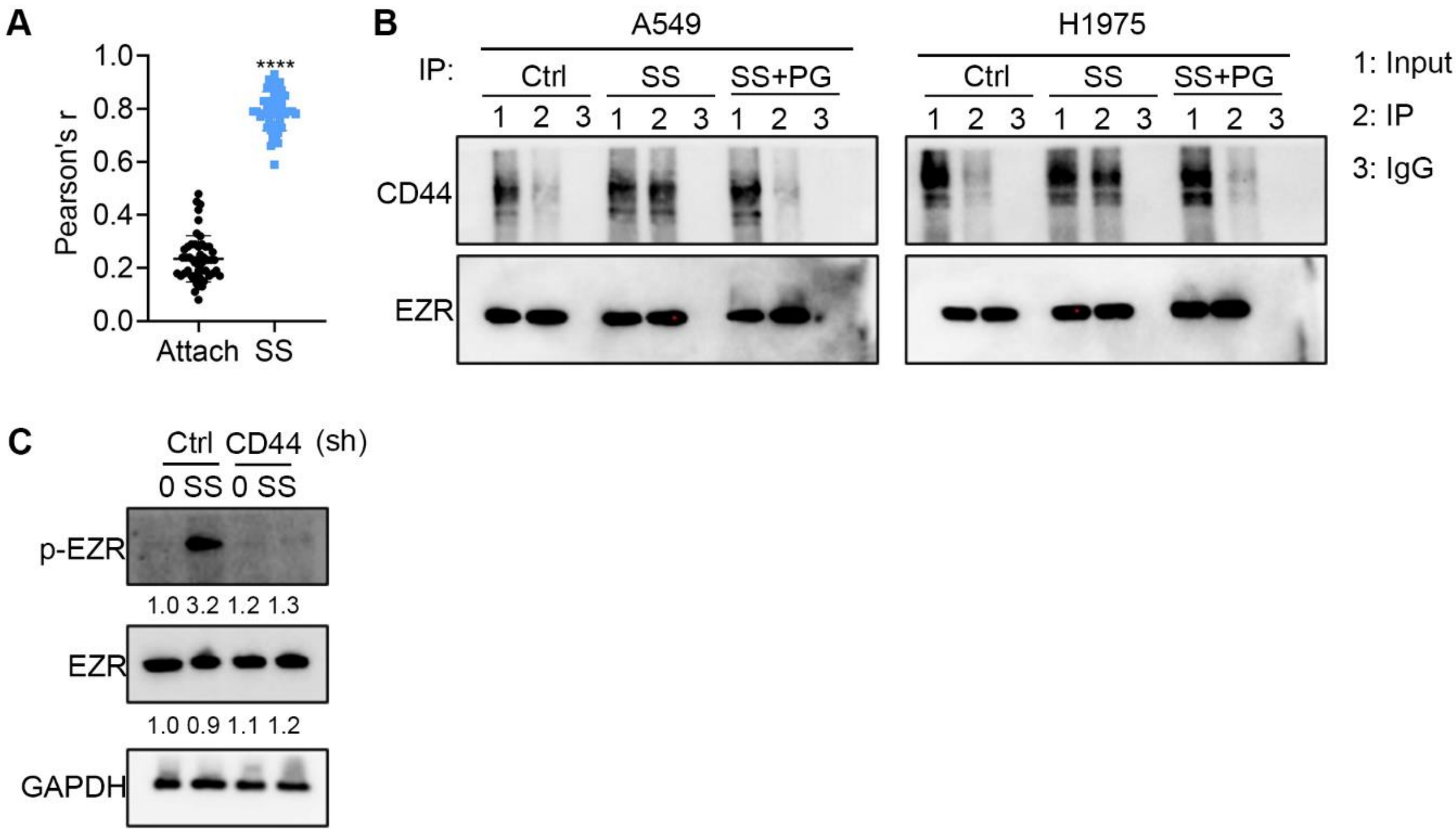


**Fig. S5.** (A) Quantitative analysis of Pearson's correlation coefficient (PCC) from 50 cells per condition (mean ± SD). Each dot represents an individual cell. ****P < 0.0001, Mann-Whitney test. (B) EZR interacts with CD44 in A549 and H1975 cells. Cell lysates were immunoprecipitated with an anti-EZR antibody (IP: EZR) or normal IgG (IgG) as a negative control. The immunoprecipitates and whole-cell lysates (Input) were analyzed by Western blotting using anti-CD44 (upper panel) and anti-EZR (lower panel) antibodies. (C) Western blot results showing the protein level of EZR and p-EZR after CD44 knockdown with or without SS treatment.

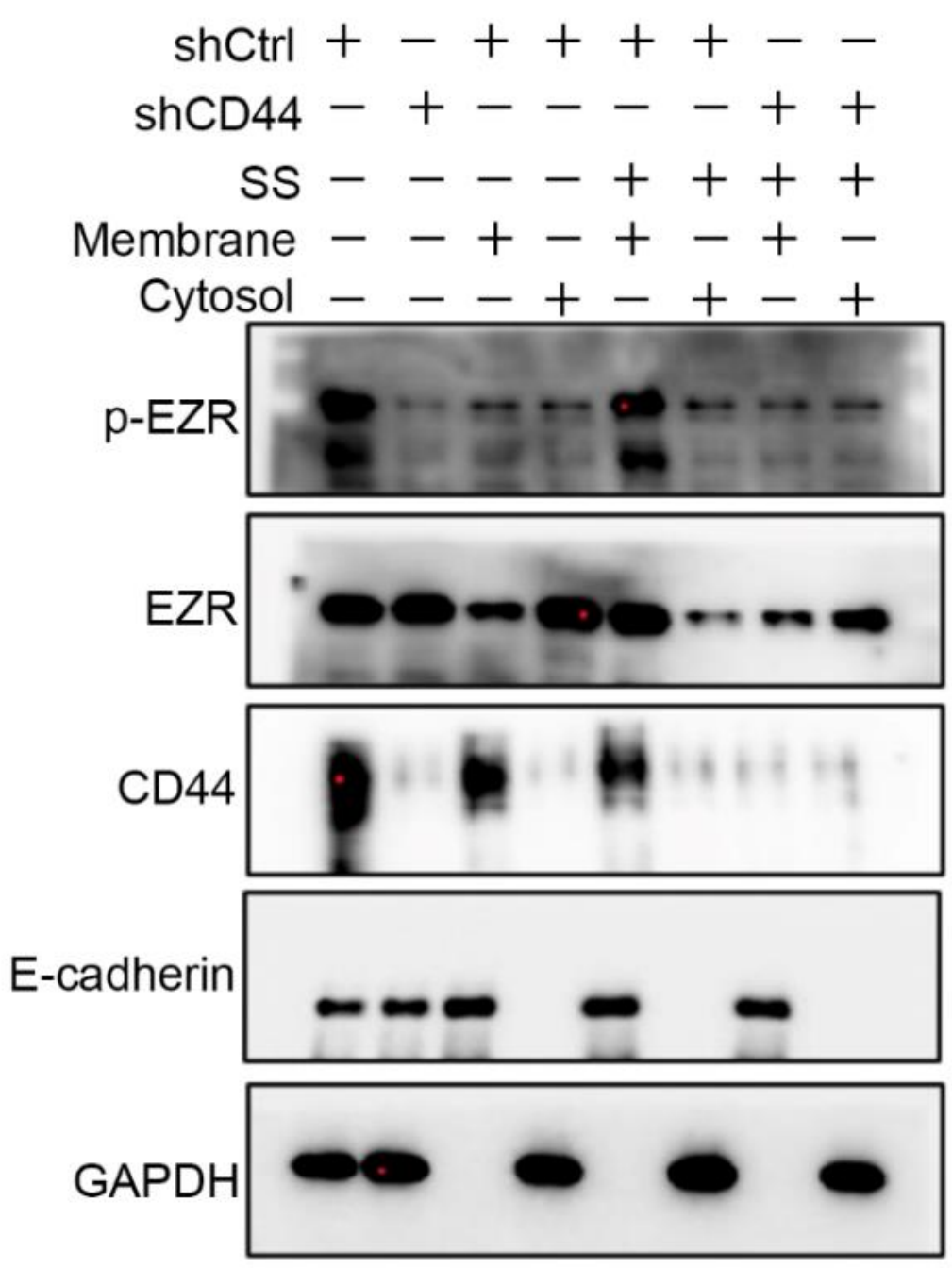


**Fig. S6.** Western blot analysis of membrane and cytoplasmic fractions from A-SSP6 cells

transfected with shCtrl or shCD44, then subjected to shear stress treatment. Fractions were probed with antibodies against p-Ezrin (Thr567), total Ezrin, CD44, and E-cadherin (membrane marker). GAPDH was used as a loading control for cytoplasmic fractions.

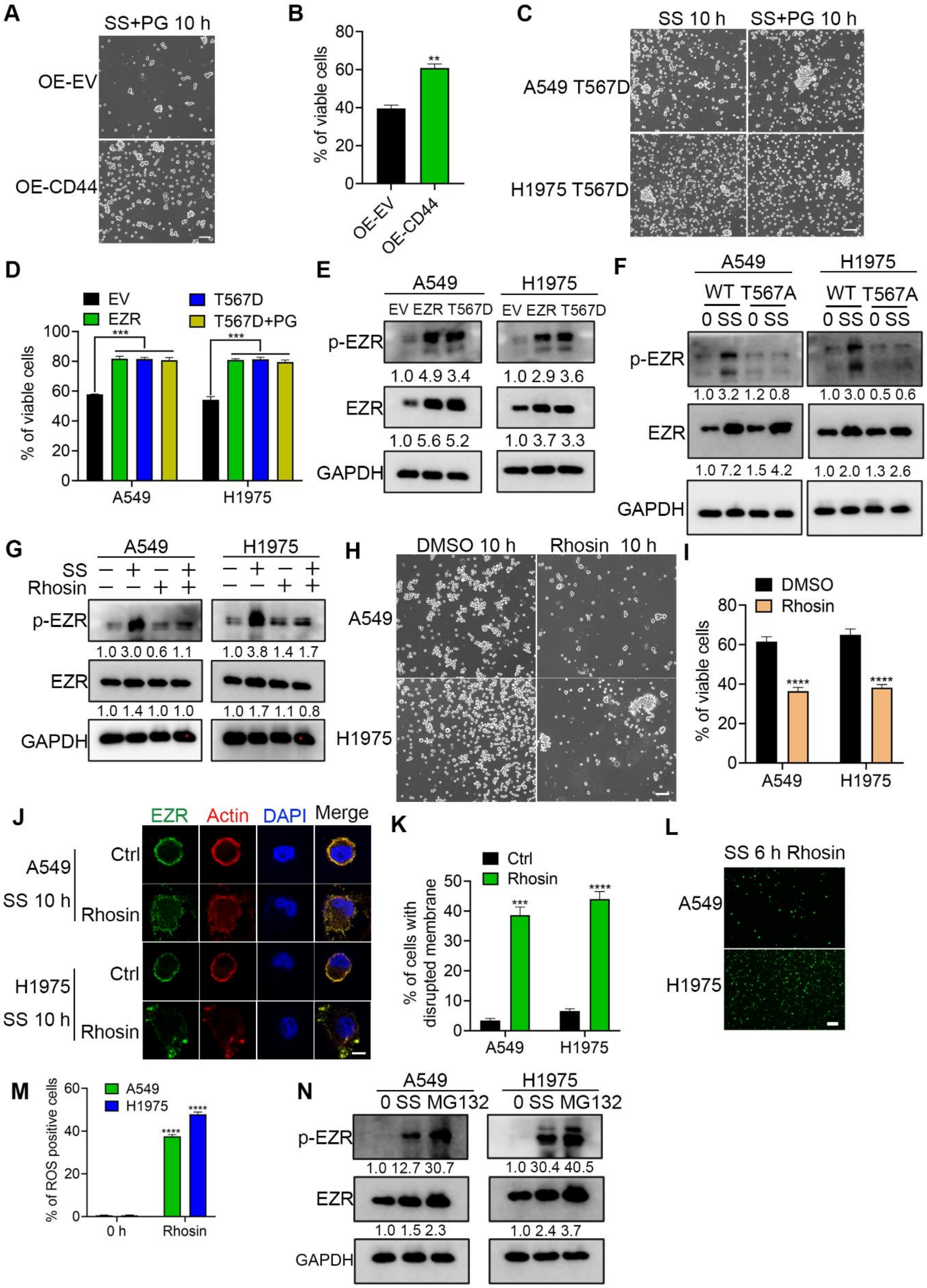

**Fig. S7.** (A, B) Representative images and quantification results of the survival rate of cells after CD44 overexpression when cells were treated with SS and PG. Scale bar: 100 µm. (C, D) Representative images and quantification results showing the SS-survival rate of cells with EZR or EZR (T567D) overexpression with or without PG treatment. Scale bar: 100 µm. (E) Western blot results showing the protein levels of EZR and p-EZR in cells with EZR or EZR (T567D) overexpression. (F) Western blot results showing the protein levels of EZR and p-EZR between wild type and T567A cells with or without SS. (G) Western blot results showing the protein levels of EZR and p-EZR after Rhosin treatment. (H, I) Representative images and quantification results of the survival rate of cells after SS treatment with or without Rhosin. Scale bar: 100 µm. (J) Representative immunofluorescence (IF) staining images showing the disrupted cell structure in A549 and H1975 cells after SS treatment with or without Rhosin. Scale bar: 5 µm. (K) Quantification result showing the percentage of cells with abnormal cell shape after circulation ($n \geq 100$ cells). (L, M) Representative images and quantification results showing the ROS levels after SS and Rhosin treatment. Scale bar: 100 µm. (N) Western blot results showing the protein levels of EZR and p-EZR after MG132 treatment. The quantification results are the means ± SD from three independent experiments. Significant differences were determined by one-way and two-way ANOVA. $^{**}P < 0.01$, $^{***}P < 0.001$, and $^{****}P < 0.0001$.

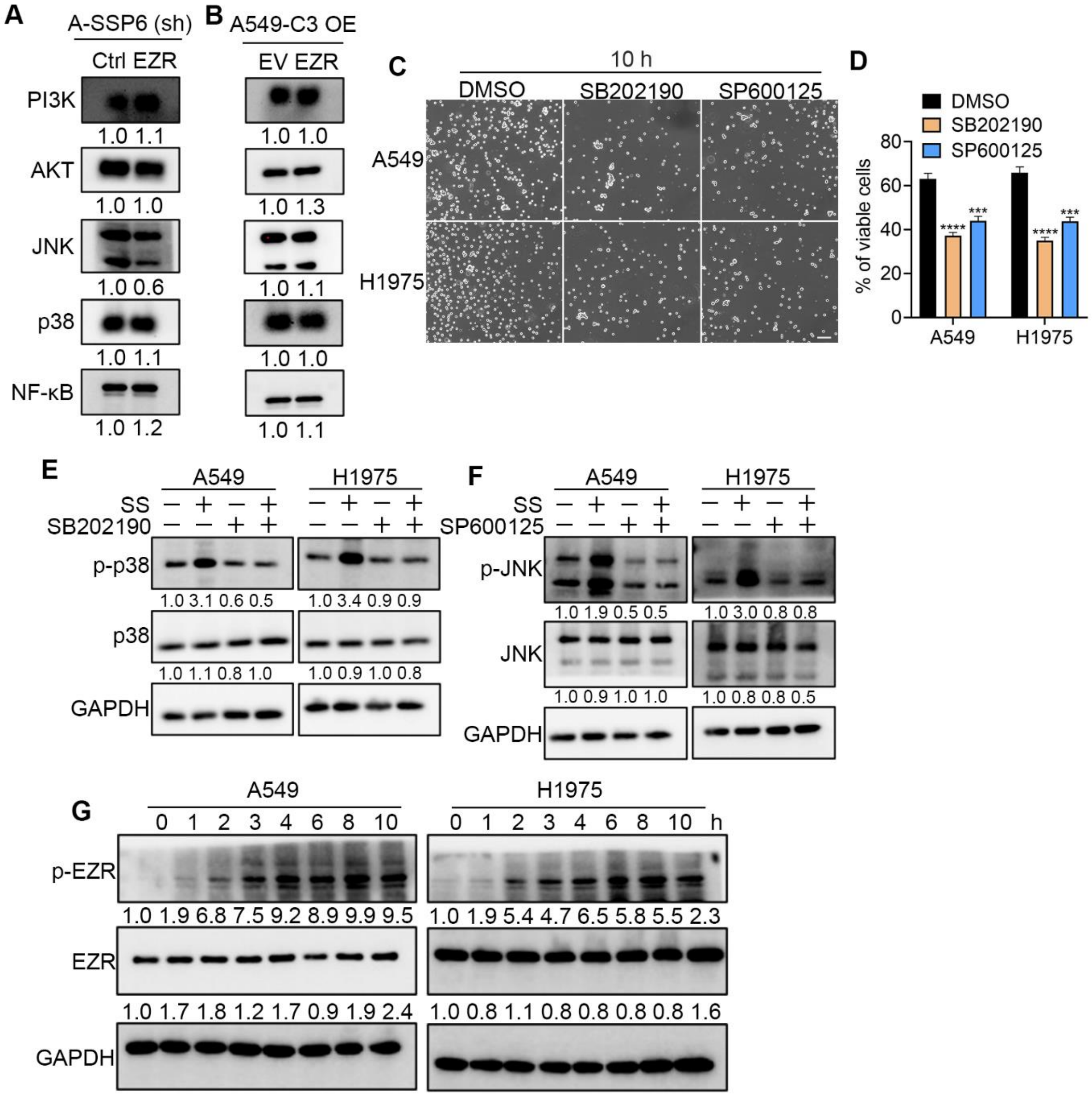


**Fig. S8.** (A, B) Western blot results showing the total protein levels of potential downstream molecules after knocking down or overexpressing EZR. (C, D) Representative images and quantification results of the survival rate of cells after p38 inhibitor SB202190 and JNK inhibitor SP600125 treatment. (E, F) Western blot results showing the protein levels of p38, p-p38 and JNK, p-JNK after inhibitors and SS treatment. (G) Western blot results showing the protein levels of EZR and p-EZR after SS treatment for different time points. The quantification results are the means ± SD from three independent experiments. Significant differences were determined by two-way ANOVA. ***P < 0.001, and ****P < 0.0001.

**Table S1. List of primers for qPCR.**

| Gene name | Forward (5’-3’) | Reverse (5’-3’) |
|---|---|---|
| GAPDH | CTGGGCTACACTGAGCACC | AAGTGGTCGTTGAGGGCAATG |
| KRT6A | GCAAATCGATCCCACCATCC | TTCTGCCTCACAGTCTTGGT |
| HAS2 | CTCTTTTGGACTGTATGGTGCC | AGGGTAGGTTAGCCTTTTCACA |
| LBH | GATTTTGACCGCTGCTGCAA | TTCGCTGTCTCTTCGCAGTT |
| EZR | ATGCCCCACGTCTGAGAATC | TCCTGCGGCGCATATACAAC |
| EMP1 | TCCACAGACCTACTGCACTG | CCAGGCTTAGCGTATTCAGC |

**Table S2. List of antibodies.**

| Antibody name | Company | Catalog # | Application |
|---|---|---|---|
| EZR | CST* | 3145 | WB (1:1000) |
| p-EZR (T567) | Invitrogen | 37763 | WB (1:1000) |
| PI3K | CST | 4249 | WB (1:1000) |
| p-PI3K p85 (Tyr458)/p55 (Tyr199) | CST | 4228 | WB (1:1000) |
| AKT | CST | 4685 | WB (1:1000) |
| p-AKT (Ser473) | CST | 4060 | WB (1:1000) |
| Bcl-2 | CST | 3498 | WB (1:1000) |
| JNK | CST | 9252 | WB (1:1000) |
| p-JNK (Thr183/Tyr185) | CST | 9251 | WB (1:1000) |
| p38 | CST | 8690 | WB (1:1000) |
| p-p38 | CST | 4511 | WB (1:1000) |
| NF-κB | CST | 8242 | WB (1:1000) |
| p-NF-κB (Ser536) | CST | 3033 | WB (1:1000) |
| GAPDH | CST | 2118 | WB (1:5000) |
| CD44 | CST | 3578 | WB (1:1000) |
| EMP1 | Abcam | 202975 | WB (1:1000) |
| Goat anti-Rabbit IgG (H+L)-HRP Secondary Antibody | Bio-Rad | 1706515 | WB (1:5000) |

| | | | |
|---|---|---|---|
| Goat anti-Mouse IgG (H+L)-HRP Secondary Antibody | Bio-Rad | 1706516 | WB (1:5000) |
| Goat anti-Rabbit IgG (H+L) Secondary Antibody, Alexa Fluor Plus 488 | Invitrogen | A11034 | IF (1:100) |
| Goat anti-Mouse IgG (H+L) Secondary Antibody, Alexa Fluor Plus 594 | Invitrogen | A11032 | IF (1:100) |

*CST: Cell Signaling Technology

**Table S3. List of shRNAs.**

| shRNA name | Target sequence (5'-3') |
|---|---|
| shEZR-1 | GCGGAGCUUGCAGAAUACACU |
| shEZR-2 | CUACAACAAAGAAGUGCACAA |
| shEMP1-1 | CGUGUUCCAGCUCUUCACCAU |
| shEMP1-2 | UUUGUUAGCACCAUUGCCAAU |
| shCD44-1 | GGAAGAAACAGCUACCCAGAA |
| shCD44-2 | ACCACACAAAACAGAACCAGG |
| shCtrl | CAACAAGATGAAGAGCACCAA |

**Table S4. List of overexpression vectors.**

| Vector name | Transcript ID | Sequence length |
|---|---|---|
| EZR | NM_003379.5 | 1761 bp |
| EMP1 | NM_001423 .3 | 474 bp |
| CD44 | NM_000610.4 | 2229 bp |
